# G3 Assisted Rational Design of Chemical Sensor Array Using Carbonitrile Neutral Receptors

**DOI:** 10.3390/s131013835

**Published:** 2013-10-14

**Authors:** Ahmad Nazmi Rosli, Maizathul Akmam Abu Bakar, Ninie Suhana Abdul Manan, Pei Meng Woi, Vannajan Sanghiran Lee, Sharifuddin Md Zain, Mohd Rais Ahmad, Yatimah Alias

**Affiliations:** 1 Department of Chemistry, Faculty of Science Building, University of Malaya, Kuala Lumpur 50603, Malaysia; E-Mails: maizathul_akmam@yahoo.com (M.A.A.B.); niniemanan@um.edu.my (N.S.A.M.); pmwoi@um.edu.my (P.M.W.); vannajan@gmail.com (V.S.L.); smzain@um.edu.my (S.M.Z.); yatimah70@um.edu.my (Y.A.); 2 NEMS & Photonics Laboratory, MIMOS Berhad, Technology Park Malaysia, Kuala Lumpur 57000, Malaysia; E-Mail: mrahmad@mimos.my

**Keywords:** sensor array design, carbonitrile neutral receptor, G3 theory, cation binding energies, hyperconjugation

## Abstract

Combined computational and experimental strategies for the systematic design of chemical sensor arrays using carbonitrile neutral receptors are presented. Binding energies of acetonitrile, *n*-pentylcarbonitrile and malononitrile with Ca(II), Mg(II), Be(II) and H^+^ have been investigated with the B3LYP, G3, CBS-QB3, G4 and MQZVP methods, showing a general trend H^+^ > Be(II) > Mg(II) > Ca(II). Hydrogen bonding, donor-acceptor and cation-lone pair electron simple models were employed in evaluating the performance of computational methods. Mg(II) is bound to acetonitrile in water by 12.5 kcal/mol, and in the gas phase the receptor is more strongly bound by 33.3 kcal/mol to Mg(II) compared to Ca(II). Interaction of bound cations with carbonitrile reduces the energies of the MOs involved in the proposed σ-p conjugated network. The planar malononitrile-Be(II) complex possibly involves a π-network with a cationic methylene carbon. Fabricated potentiometric chemical sensors show distinct signal patterns that can be exploited in sensor array applications.

## 1. Introduction

**Scheme 1.**
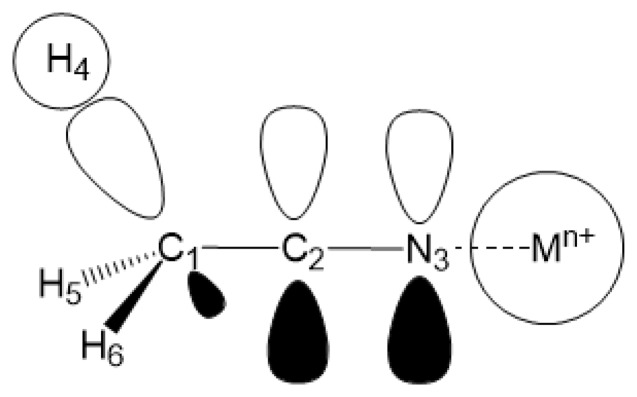
*σ*-*p* Conjugation stabilized acetonitrile-cation complex.

Carbonitriles can be synthesized by a variety of preparative procedures and the functionality can be further transformed into numerous functional groups [1]. The cyano functional group appears as a synthetic equivalent in useful carbon-carbon formation steps [2–5]. However this synthetically feasible functionality has rarely been considered for detection of ionic species. In this work we describe combined computational and experimental strategies in exploiting unconjugated carbonitrile moieties for chemical sensor array applications [6,7]. Acetonitrile is a simple model for unconjugated carbonitrile receptor and extensively analyzed in this work. The carbon-carbon bond in acetonitrile is much shorter compared to that in ethane [8], and this has been attributed to σ-conjugation that could facilitate binding to metal cations [9] (Scheme 1).

Electrochemical sensors such as ion selective electrodes (ISEs) [10] and chemically modified field effect transistors (ChemFETs) [11–13] are the most commonly employed devices for detecting ionic analytes. In these sensors, chemical activity is converted to a voltage signal using a reversible reduction-oxidation electrode such as silver-silver chloride or doped conducting polymer [14]. The sensor signal is baselined to a reference electrode, typically a double-junction silver-silver chloride electrode having a constant chloride concentration and suitable external electrolytes. A common sensor error arises from false signals due to interfering ions. Only when the sensor selectivity is very high and in the presence of low concentrations of interfering ions, this type of noise can be neglected [15]. In cases where the analyte is surrounded by high concentrations of interfering ions, a high selectivity sensor will no longer give reliable measurements. The situation is even worse when the required linear range is narrow or the limit of detection is at the sub-ppm level.

Selectivity requirement for chemical sensors depends on the intended target application. Our electrochemical sensors primarily target ionic analytes in agriculture, aquaculture, environmental monitoring and medical applications. Linear range and accuracy requirement for the same analyte differ tremendously across verticals. For example nitrate sensor in precision agriculture normally monitors the nutrient in the range of 100 to 300 ppm, whereas, for nitrate-poisoned carrot detection capability at less than 10 ppm is more appropriate. A more striking difference in requirements can be seen in dissolved ammonia sensors. The requirement for monitoring dissolved ammonia in industrial discharge before treatment is at tens of ppm, whereas, in aquaculture application, unionized ammonia is considered lethal at sub-ppm levels [16].

Here in this paper, we employ computational method in designing neutral receptor molecules for charged analytes. Our target applications require detection of sodium, potassium, magnesium, calcium, ammonium, nitrate, phosphate, chloride and sulfate. In sensor array approach the burden on achieving very high selectivity towards the target analyte is relieved because the accuracy is no longer determined by sensor selectivity alone. In fact, the main challenge in sensor array approach is the ability in recognizing signal patterns produced by different analyte concentrations in the presence of interfering ions.

Neutral receptor molecules (ionophores) typically exploit organic functional groups to achieve binding with target analytes [17,18]. In cation recognition, electron lone-pairs on heteroatoms, carbonyl groups and heterocycles are often used, whereas for binding to anions, different types of hydrogen bonds are utilized [19]. In cation recognition, the combination of heteroatom lone pairs and ring cavity size could discriminate metal cations based on ionic radius size. This strategy does not work for anions, where the Hofmeister effect dominates selectivity trend [20]. Selective detection of neutral molecules is much more challenging and enzymes give the most reliable results. Synthetic receptors for small neutral molecules need to use combinations of the already mentioned strategies including complex cavity shape or molecular imprint [21,22].

## 2. Results and Discussion

### 2.1. Computational Benchmarks

Accuracy of the adopted computational methods has been benchmarked with receptor-analyte binding models. The benchmarks compare the performance of the above computational methods with each other and whenever available, with experimental values. The benchmark models illustrate simple cases relevant to interactions between chemical sensor and target molecules.

The geometry, vibrational frequencies and binding energy of water dimer represent a simple and useful benchmark for receptor-analyte weak interaction involving hydrogen bonding. The experimental distance between the two water molecules measured as the distance between two oxygen atoms (*r*_o-o_) is 2.978 Å (Table 1) [24,25]. The G3 and MQZVP methods give comparable *r*_o-o_ (2.913 Å and 2.918 Å, respectively) and close to the experimental value, whereas, the separation in CBS-QB3 is considerably shorter (3%). Similarly, the G3 and MQZVP vibrational frequencies for hydrogen bonding are closer to each other while CBS-QB3 predicts the interaction to be stronger by 20 cm^−1^. All three computational methods are able to reproduce experimental dissociation energy of water dimer within 1 kcal/mol.

Donor-acceptor type interaction is another well-known computational benchmark that is applicable to chemical sensor design. Optimized structures of ammonia-borane complex, nitrogen-boron bond distances, vibrational frequencies and dissociation energies of the complex were calculated using G3, CBS-QB3 and MQZVP methods (Table 2). Available experimental data are provided for comparison purposes [26,27].

Due to the stability of the ammonia-borane adduct in water, the respective results in water media are also provided. The three methods afford practically the same gas-phase boron-nitrogen bonds that reproduce the experimental bond length determined by neutron diffraction method on NH_3_-BH_3_ crystals [29]. In water, the *r*_B-N_ values are consistently shorter and practically identical. The G3 and CBS-QB3 gas-phase dissociation energies are quite comparable, that is about 3 kcal/mol higher than predicted by MQZVP method. The dissociation in water follows similar trend, with MQZVP gives 3 kcal/mol lower. The shorter *r*_B-N_ bond lengths and higher D_e_ in water strongly suggest greater stability of the ammonia-borane pair in water, presumably due to stabilization of the ionic complex in polar solvents. The three methods predict the same value (1.21 Å) for nitrogen-hydrogen bond distances, and there is no solvent effect on *r*_B-H_ values. However, the neutron diffraction measurement reported a shorter bond length (1.15 Å). Likewise, all the predicted nitrogen-hydrogen bond lengths are almost identical (1.02 Å), and again there is no solvent effect. The experimental *r*_N-H_ measured by neutron diffraction method is slightly less than 1 Å. MQZVP and CBS-QB3 predicts boron-nitrogen stretching frequencies of approximately 640 cm^−1^, 40 cm^−1^ higher than G3 prediction. The computed N-B stretching in water is about 80 cm^−1^ higher consistent with a stronger interaction and shorter bond distance. Furthermore, the ammonia-borane binding energies are in keeping with the predicted geometries. The three methods show that the ammonia-borane complex is strongly bound, and the interaction is more than 5 kcal/mol stronger in water. The energy predicted by MQZVP is 3 kcal/mol lower compared to the G3 and CBS-QB3 energies. If this benchmark can be generalized to other donor-acceptor systems, MQZVP method is expected to predict lower binding energies.

Another important benchmark useful in neutral receptor design is the interaction of electron lone pairs on heteroatom to metal cations. Interactions of Mg(II) ion with water and hydrogen cyanide in the gas phase are compared by employing the G3 and G4 methods on the lone-pair-cation system (Table 3).

The binding energies differences between the G3 and G4 methods, for both cases (H_2_O and HCN), are less than 1 kcal/mol. The G4 Mg-N and Mg-O bonds are shorter and consequently the stretching frequencies are larger with the G4 method. The C-N and O-H bonds produced by the two Gn methods differ by about 0.01 Å.

### 2.2. Acetonitrile Carbonitrile Simple Receptor Model

**Figure 1.**
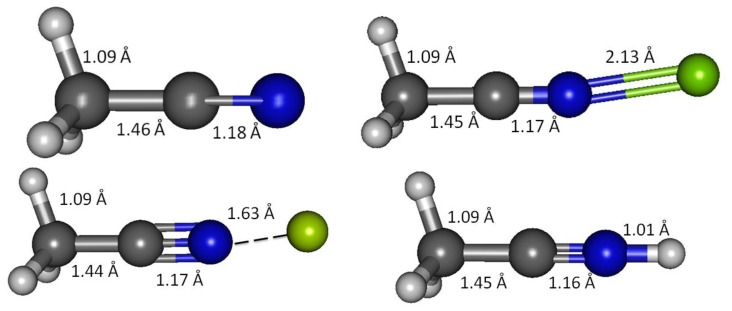
G3 optimized geometries of acetonitrile and its complexes with Be(II), Mg(II) and H^+^. (**i**) CH_3_CN; (**ii**) CH_3_CN-Mg(II); (**iii**) CH_3_CN-Be(II); (**iv**) CH_3_CN-H^+^.

Acetonitrile lacks a *π*-conjugation system and serves as a simple model of an unconjugated carbonitrile. Geometry optimizations in vacuum and solvated media were initially explored using the B3LYP method and 6-31 + G(d,p) basis set. Subsequently G3, G4, CBS-QB3 and MQZVP methods were used to obtain structures and energies at these levels of theory. The C1-C2 bond distance, predicted by all computational methods are apparently shorter (1.460 Å, G3; Figure 1 and Table 4) compared to the carbon-carbon single-bond in ethane (1.532 Å, B3LYP) [23]. These results are in good accord with experimental bond distances that report shorter carbon-carbon bond distances in acetonitrile (1.458 Å) and butadiene (1.476 Å), compared to that in ethane (1.536 Å) [8]. Shortening of the carbon-carbon bond in butadiene by 4% has been attributed to *p*-*p* conjugation, widely known as hyperconjugation [34]. Practically this refers to interaction of two 2*p*_z_ orbitals at C2 and C3 in butadiene that improves the entire *π*-network. This is supported by preference for adoption of planar conformation that maximizes the overlapping of adjacent 2*p*_z_ orbitals [9,35,36]. Likewise, as depicted in Scheme 1, the shrinking of the C1-C2 bond in acetonitrile by close to 5% compared to ethane (B3LYP) [23] can be attributed to *σ*-*p* interaction similar to the hyperconjugation reasoning, described earlier [37].

The G3 C2-N3 bond distance in acetonitrile is 1.178 Å, about 2% longer than the experimentally determined value (1.157 Å) [8]. However, experimental C-N bond in hydrogen cyanide (1.156 Å) [8] is practically identical to that in acetonitrile. This indicates that the cyano functional group retains the essential carbonitrile triple bond and lone-pair character regardless of the neighbors. Consequently, in weak interactions of interest to chemical sensor design and function, the C-N multiple bond in carbonitrile most probably is inaccessible, and thus the interaction is primarily determined by the cyano lone-pair electron and the nature of the positively charged analytes (this work focuses on Ca(II), Mg(II), Be(II) and H^+^).

According to the *σ*-*p* conjugation model, the shrinking of the C1-C2 bond in acetonitrile could possibly increase the bond order, and discriminate the hydrogen atoms attached to C1. The C1-H bond distances should no longer be identical. Analyzing the three C-H bond distances at four decimal places reveal the following bond lengths; C1-H6 (1.0910 Å) and C1-H5 and C1-H4 (1.0911 Å both). However, when rounded to three decimal places, the optimized geometries at all levels of theory, give the same C1-H bond distance for the three C-H bonds (1.091 Å, G3 water) and practically identical H-C1-H bond angles (109.9°, G3 water). Moreover, GIAO ^1^H-NMR simulation (*versus* TMS B3LYP/6-311 + G(2d,p) reference) on uncomplexed acetonitrile receptor using the B3LYP optimized structure affords a single peak at 2.28 ppm (Table 5) for the three protons. The GIAO ^13^C NMR (*versus* TMS B3LYP/6-311+G(2d,p) reference) gives −8.72 and 103.77 ppm for C1 and C2, respectively, consistent with a normal *sp*^3^ and *sp* hybridized carbon nuclei [38].

The Mulliken charges on uncomplexed acetonitrile (Table 6) is consistent with the expected electronegativity of the atoms. Nitrogen atom being the most electronegative carries the most negative charge of −0.516. C2 which is directly attached to nitrogen loses significantly its electron density to N3, resulting in a net of 0.295 Mulliken charge. Holding three hydrogen with 0.106 Mulliken charge, C1 has a normal small negative charge of −0.097.

The C1-C2 stretching frequency in acetonitrile, computed by the B3LYP method is 924.8 cm^−1^, while the C-C stretch in ethane [39] gives 999.36 cm^−1^ (Table 7). The results indicate that C-C single bonds in both cases are essentially the same. A higher frequency is expected for acetonitrile if the proposed *σ*-*p* conjugation results in substantial barrier to C-C rotation and strong orbital (2*p*_z_) overlap. In keeping with the hyperconjugation reasoning, the computed cyano C2-N3 stretch in acetonitrile is 2,586.1 cm^−1^ (RHF/6-31 + G(d,p)), 148 cm^−1^ higher than that in hydrogen cyanide (2,438.2 cm^−1^, G3) [23].

**Figure 2.**
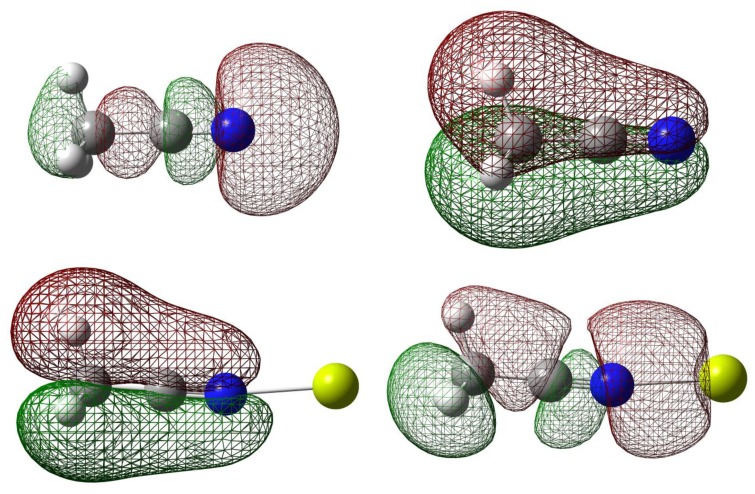
Selected G3 molecular orbitals of acetonitrile and its complexes with Mg(II). (**i**) HOMO-2 of CH_3_CN; (**ii**) HOMO-3 of CH_3_CN; (**iv**) HOMO-3 of CH_3_CN-Mg(II); (**v**) HOMO-4 of CH_3_CN-Mg(II).

Natural Bond Orbital (NBO) can help elucidate the effect of orbital occupancy on the structure and reactivity of the carbonitrile receptor, as well as the relative stability of the receptor-analyte complexes [40,41]. The change in *s versus p* character in chemical bond of interest usually gives rise to significant difference in structure and reactivity. In chemical sensor design, orbital occupancy can be used to explain delocalization of valence electron clouds through some sorts of resonance networks, either exclusively *π*-bonds or mixture of *σ*- and *π*-bonds. The orbital occupancy data are also intuitively connected to molecular orbitals and their energy levels in order to assign frontier orbitals involved in receptor-analyte binding (Figure 2). For example, in assigning the orbital for lone-pair electron, orbital degeneracy suggests that it is safe to assign these equal energy MO's to two *π*-bonds thus the lower energy *sp* hybrid MO is more suitable for a lone pair (*i.e.*, the HOMO-2 in acetonitrile).

In acetonitrile the C1 carbon has a lower 2*p*_x_ (1.08) occupancy compared to the 2*p*_y_ and 2*p*_z_ orbitals (both 1.30), while the 2*s* occupancy is 1.14 (Table 8). All the C1 orbitals have occupancies in excess of 1 because of the contribution of the three hydrogen atoms attached to C1. Likewise the occupancy of the H orbitals are all less than 1 (0.699), after donating to the more electronegative C1. On the contrary, all C2 orbitals have occupancy less than 1 [2*p*_x_ (0.99); 2*p*_y_ and 2*p*_z_ (both 0.88); 2*s* (0.89)], giving up to the more electronegative nitrogen neighbour. The 2*p*_y_ and 2*p*_z_ occupancies in C2 are consistently the same, suggesting that these 2*p* orbitals are used to form two *π*-bonds in the carbonitrile triple-bonded functionality. Consequently, the 2*s* and 2*p*_x_ orbitals are used by C1, C2 and N3 to form *σ*-bonds, and one is also available to accommodate a lone-pair electron on N3. The *s* and *p* characters of the *σ*-bonds of interest are consistent with these occupancy data, while essentially all *π*-bonds are constructed with pure 2*p* orbitals (Table 8). The C1-C2 *σ*-bond in acetonitrile uses typical *sp*^3^ orbitals for bonding having close to ideal 24% *s* and 76% *p* characters on C1, and employs close to ideal *sp* hybrid orbital from C2 (54% *s* and 46% *p* characters, Table 9). Likewise, the C2-N3 *σ*-bond shows close to ideal *sp* hybridized orbitals (46% *s* and 54% *p* on C2, and 45% *s* and 55% *p* on N3—the *sp* orbital having more *p* character in uncomplexed acetonitrile.

### 2.3. Acetonitrile-Mg(II) Complex

Acetonitrile approaches and interacts with positively charged analytes through its lone-pair electrons. Unlike olefins, where protonation involves a direct interaction between hydrogen and the 2*p* orbitals in the *π*-bond, resulting in a new C-H *σ*-bond and carbocation. The breaking of the cyano C-N *π*-bond of acetonitrile is not feasible due to the formation of an unstable vinyl cation [42–46]. In protonated olefin, the resulting cation occupies a 2*p*_z_ orbital, having three substituents in the x-y plane that can interact and stabilizes the positively charged carbon center. On the contrary, the empty orbital in vinyl cation is on the same plane with the double bond and not stabilized by the neighbours [1,9].

**Figure 3.**
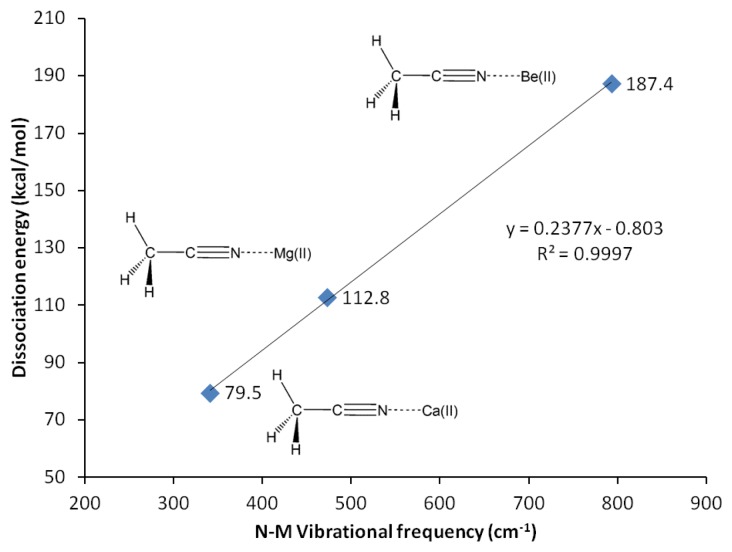
G4 Dissociation energy *versus* N-M vibrational frequency (in vacuum).

G3 calculations indicate that divalent magnesium cation is bound to acetonitrile in water with a binding energy of 12.5 kcal/mol (Table 10). Using the same method, the ammonia-borane benchmark shows three-fold stronger binding in water (35.1 kcal/mol, Table 2). The binding energy calculations have been repeated using G4 method in order to include calcium(II) and allow plotting of Figure 3. The C2-N3 *σ*-bond in acetonitrile-Mg(II) complex shows significant increase (18%) of the *s* character at the *sp* orbital used to accommodate nitrogen lone-pair electrons, now forming bonding with magnesium(II), compared to the uncomplexed receptor model (increased from 45.1 to 53.2% *s* character, Table 9). On the contrary, as the N3 increases its s character in order to optimize overlapping with Mg(II), C2 increases its *p* character by 3% (Table 9). The 2*s* and 2*p*_x_ orbitals in C2 and N3 are involved in the C2-N3 *σ*-bond. Interaction with Mg(II) presumably is limited to these orbitals, as the binding process would avoid forming vinyl cation [42–46].

The interaction with Mg(II) causes reduction of 10% of the occupancy in the C2 orbitals involved in *π*-bondings, but negligible difference in the 2*p_x_* orbital used for *σ*-bonding. On the contrary, the interaction with magnesium cation causes N3 to receive 8% additional occupancy in the 2*p* orbitals for *π*-bonding, and 5% increase in the occupancy for *σ*-bond. This balance would possibly leave the C2-N3 *π*-bonding characteristics unchanged. Moreover, the combined C2 and N3 2*s* occupancies in the uncomplexed and the complex after binding with Mg(II) remains essentially the same (2.4, Table 8). As a whole, the interaction with Mg(II) affords no noticeable change in *π*-bonds characteristics, whereas, the observed shortening of the C2-N3 triple bond could be attributed to a more effective *σ*-bond that accompanies significant increase in N3 *s* character (18%, Table 9).

As acetonitrile interacts with magnesium cation, the C1-C2 bond becomes shorter—G3 calculations show 1.4% shortening of the bond (shrinking from 1.460 Å to 1.453 Å, Table 4). The shortening of C2-N3 due to the interaction is less than 1% (from 1.168 Å to 1.170Å, Table 4). Effectively, interaction with magnesium shortens both bonds. When rounded to three decimal places, all the three C1-H bonds remain essentially unchanged (1.091 Å).

**Figure 4.**
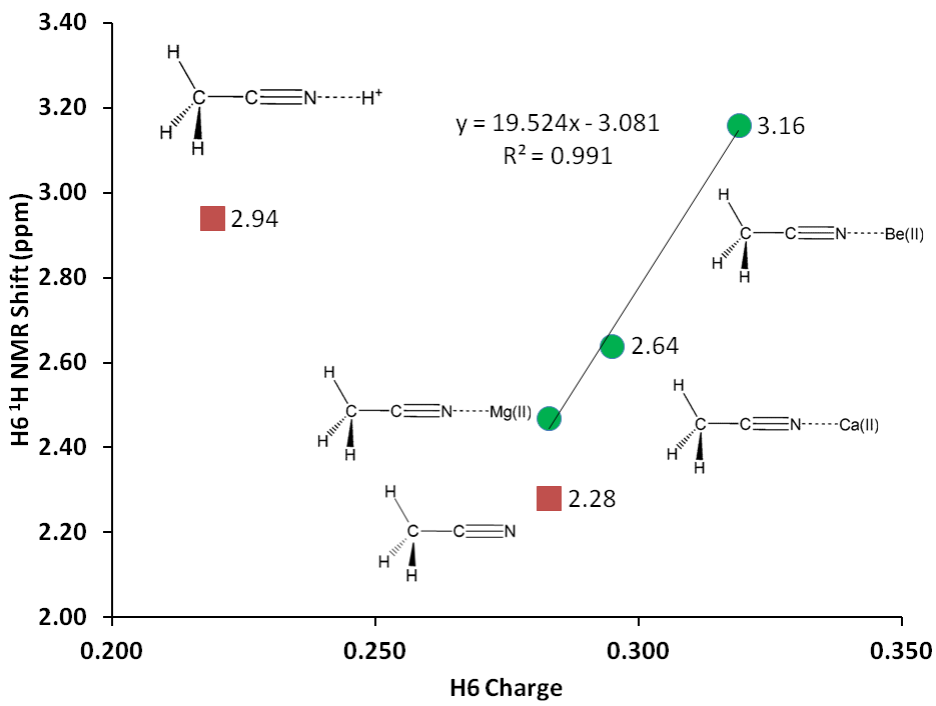
H6 ^1^H-NMR shift *versus* H6 charge (G3, vacuum).

^1^H-NMR of the three H atoms attached to C1 provides valuable probe to monitor changes in C1-H bond due to *σ*-p conjugation in acetonitrile-analyte complex. Likewise, NMR shifts of the C1, C2 and N3 nuclei could elucidate changes in electron clouds shielding these nuclei. Despite insignificant change in the C1-H bond distance (1.091 Å), the H atoms attached to C1 experience significant deshielding effects due to interaction with Mg(II). The interaction with magnesium cation helps discriminate the H nuclei giving two types of H one H in the x-z plane and two H's at x-y plane. The two types of H are deshielded by close to 30% due to the interaction with Mg(II) (Table 5, Figure 4). Similarly, direct interaction with Mg(II) significantly causes shielding on the N3 nuclei—the N3 NMR shift reduces by about 20%, in keeping with a buildup of electron density surrounding the N3 nuclei. Logically, a shift of electron density from C2 to N3 could in turn deshield C2, and as expected a 6% increase in C2 NMR shift is observed. The deshielding effect on both carbon nuclei occurs at the same extent as C1 also records an increase of 6% in NMR shift.

In binding with acetonitrile receptor, magnesium cation transfers 0.246 of its charge to the receptor. The changes in Mulliken charges due to this interaction are the most noticeable—the key questions here are; (i) How does acetonitrile distribute the 0.246 of plus charge it receives from Mg(II)? and (ii) Which atom(s) receive most of this charge? The charge at nitrogen has become less negative by 73% due to direct contact with magnesium cation, and each of the H atom are more positively charged by 17% apparently these account for almost all of the imparted charge from magnesium. The structural changes in the compex (compared to the uncomplexed acetonitrile) can be explained in terms of redistribution of the imparted charge from Mg(II) in order to sustain interaction with the analyte. For example, C1 undergoes a huge increase in Mulliken negative charge by 273% to balance the highly positive hydrogen atoms. This is in good agreement with the observed deshielding of these nuclei. Sandwiched by and in direct contact with highly negative C1 and strongly positive N3, C2 undergoes a net increase in positive charge by 27%. This effectively leaves the cyano group as a positively charged functional entity.

The errors in theoretical vibrational frequencies, computed using different methods, could be in tens of cm^−1^. Therefore, it is not considered reliable to use small differences in observed stretching frequencies to probe structural changes [47] thus information from experimental FTIR measurement is more appropriate for chemical sensor design. Nevertheless, computational methods correctly confirm earlier structural observations that interaction with Mg(II) increases overlap in bonding orbitals between C1 and C2 and between C2 and N3, leading to the prediction of higher stretching frequencies and shorter bond distances. Table 7 shows increase of C1-C2 stretching frequency from 954.8 cm^−1^ to 966.7 cm^−1^ and C2-N3 triple bond vibration from 2,586.1 cm^−1^ to 2,620.8 cm^−1^.

**Figure 5.**
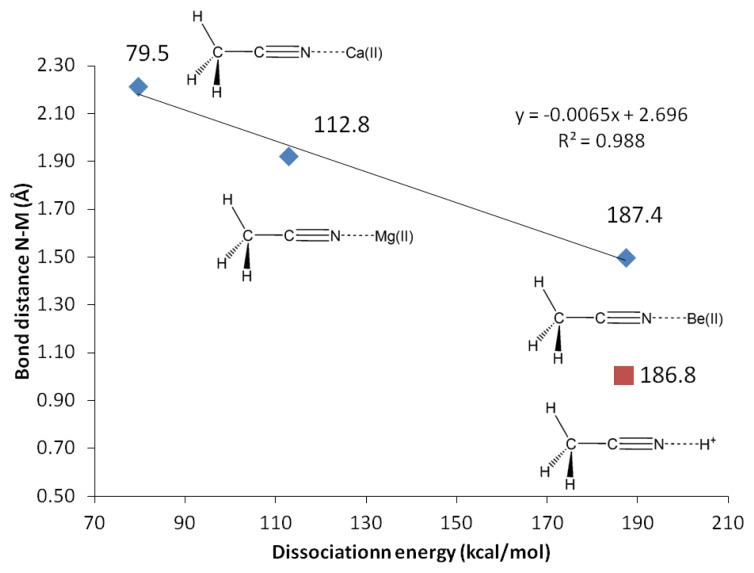
N3-Cation bond distance *versus* acetonitrile binding energy (G4, vacuum).

In actual chemical sensor applications, it is more important to compare magnesium cation binding to that of calcium, another divalent cation analyte of significant importance in metabolite detection. However, our attempts using all computational methods lead us to conclude that acetonitrile is bound to calcium only in vacuum (Table 10, Figure 5). The characteristics of solvated receptor-analyte interaction especially in water medium is much more important, whereas Ca(II) is presumably not bound to unconjugated carbonitriles in water medium. In a separate report [48] we demonstrate that conjugated carbonitrile is strongly bound to magnesium cation in aqueous medium.

### 2.4. Acetonitrile-Be(II) Complex

Beryllium(II) is much more strongly bound to acetonitrile compared to its Group II neighbors; magnesium(II) and calcium(II). The three methods employed, G3, CBS-QB3 and G4 are in good agreement in showing that the binding energy of Be(II) to acetonitrile is three fold as strong compared to Mg(II) (12.5 kcal/mol with Mg(II) compared to 42.3 kcal/mol with Be(II), Table 10). In general, beryllium(II) and magnesium(II) show consistent trends in all the structural and reactivity parameters analyzed in this work.

NBO analysis reveals that Be(II) affects the orbital occupancy in all the atoms and bonds in acetonitrile to greater extents compared to the effects by Mg(II) described earlier. After binding with beryllium(II) cation, the cyano N3 becomes more electronegative, as can be seen in the decrease of the occupancies of 2*p*_y_ and 2*p*_z_ orbitals in C2, directly attached to N3 (17% decrease from uncomplexed acetonitrile), and a reduction of 3% in the 2*p*_x_ occupancy (Table 8). On the contrary, the change in 2s orbital occupancy in C2 is negligible.

The electron density is transferred to N3 that experiences a 15% increase of its 2*p*_y_ and 2*p*_z_ orbitals. Unlike C2 that retains its 2*s* electron, N3 2*s* orbital loses 17% of its electron population while forming bond with Be(II). In similar trend with Mg(II) but in greater extents, binding with Be(II) causes significant changes in the *s versus p* character of the cyano functionality. While in uncomplexed acetonitrile the C2-N3 sigma bond is essentially constructed using close to 50/50 of *s*/*p* character making it an ideal *sp* hybrid orbital, the occupancy is now changed to 40/60 in C2 and 60/40 in N3 (both *s*/*p* in % characteristics, Table 8). Likewise, the 2*p*_y_ and 2*p*_z_ orbitals in C1 receive higher (1%) occupancy compared to uncomplexed acetonitrile. The 2*p*_x_ orbital in C1 undergoes a 4% reduction of its occupancy while the 2*s* orbital occupancy increase by 2%, compared to uncomplexed acetonitrile. The net result after the give-and-take between C2 and N3 in the C2-N3 *π*-bond, and between the *s versus p* in the C2-N3 *σ*-bond could possibly leave the cyano triple bond unchanged. The optimized acetonitrile-Be(II) structure shows that both C1-C2 and C2-N3 bonds are shorter compared to those in acetonitrile (Table 4). The shortening of the C1-C2 bond could be attributed to *σ*-*p* conjugation. The shrinkage of the cyano bond distance presumably due to a stronger C-N bond either more efficiently rehybridized *sp* orbitals in the *σ*-bond alone or with additional contribution from more efficient *π*-bonds.

Beryllium cation transfers 0.75 of its positive charge to acetonitrile and the impact of beryllium cation on N3 is so significant that in the complex its Mulliken charge has become positive [from −0.516 in acetonitrile to 0.212 in the Be(II) complex]. Interestingly, the Mulliken charge analysis provides the only example where the beryllium effect on C1 is less significant compared to that of Mg(II) – C1 becomes less electronegative in the former. Moreover, Be(II) effect on C2 is also less compared to that of Mg(II). However, the Mulliken charges of the H atoms increase by close to 30% compared to uncomplexed acetonitrile, and the 2 H nuclei separated even further.

In similar trend with Mg(II) but to a greater extent, the Be(II) binding affords noticeable changes on the vibrational frequencies. G3 C1-C2 stretching frequency is increased by 102 cm^−1^ in the Be(II) complex, whereas the increase in the C2-N3 triple bond stretch is only 37 cm^−1^. The Be-N stretch is at 728 cm^−1^, about two-fold compared to Mg-N stretching frequency (Table 7). Be(II) effects on C1 and C2 nuclei are similar to the effects of Mg(II), with only slightly higher deshielding effects (Table 5, Figure 4). However, the change on the three H nuclei due to Be(II) is much more significant –30% increase in ^1^H-NMR shifts and the two types of H are even more separated (by 0.04 ppm).

### 2.5. Protonated Acetonitrile

Protonated acetonitrile does not follow the general trends of either the Group I or the Group II cation analytes. With dissociation energy of 138.2 kcal/mol, proton is bound to acetonitrile more than ten times as strong, in comparison to the binding energy in CH_3_CN-Mg(II) (Table 10, G3). In protonated acetonitrile, the C2-N3 bond is shortened even further (1.155 Å) compared to those in the magnesium and beryllium complexes a reduction of 2% compared to the cyano bond in acetonitrile. Likewise, the C1-C2 bond in the protonated complex is shortened by 1% (Table 4). The protonation of acetonitrile does not lead to significant change to the C1-H bond length (Table 4). However, there are noticeable changes in the H-C1-H bond angles (108.7° and 110.2°, Table 4). The protonation of acetonitrile does not affect the Mulliken charge on C1. On the contrary, the changes on C2 and N3 Mulliken charges are large –C2 experiences an increase in Mulliken charge by 0.097 and N3 becomes less negative by 0.421. By forming the protonated complex, the proton imparts 0.632 of its charge, and almost all of it is received by N3 (Table 6). NBO analysis shows the C2-N3 *σ*-bond has more *s* character in N3 while the *s* and *p* characters at C2 are essentially the same as in the uncomplexed receptor. The C1-C2 *σ*-bond orbitals show no noticeable change in the uncomplexed and protonated receptor—the C2 orbital is almost ideal *sp* hybrid orbital constructed with 50/50 *s* and *p* and the C1 is a typical *sp*^3^ hybrid orbital (Table 9). The ^1^H nuclei in the protonated acetonitrile are significantly deshielded (by 0.66 ppm or an increase of 22%) but there is only one type of H nuclei, whereas, in striking contrast to Mg(II) and Be(II) complexes, both the C1 and C2 nuclei are shielded (Table 5). Consistent with the observed shortening of the cyano C-N bond distance, protonation affords a slightly higher stretching frequency (2,609.0 cm^−1^). The N3-H stretching frequency (3,938 cm^−1^) indicates a very strong bond, in keeping with the observed short bond distance (1.014 Å, Table 7).

### 2.6. n-Pentylcarbonitrile-Mg(II) Complex

**Figure 6.**
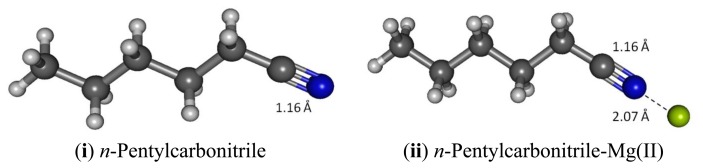
Optimized geometries of *n*-pentane carbonitrile and its complex with Mg(II).

Lipophilicity is an essential physical characteristic of membrane-based sensing layer. Long hydrocarbon chains are typically employed to make a recognition molecule more compatible with the membrane—small molecules tend to leach out from the solid phase during long exposure to aqueous environment. *n*-Pentane is considerably a better model for a lipophilic carbonitrile in terms of structure and reactivity, compared to acetonitrile, and is still computationally feasible. Our results confirm that in all key parameters investigated, *n*-pentylcarbonitrile, carbonitrile and acetonitrile essentially show identical characteristics. In keeping with the observed C1-C2 bond in acetonitrile, B3LYP calculation gives a bond distance of 1.460 Å, apparently shorter (Figure 6) compared to the carbon-carbon single-bond in ethane (1.532 Å, B3LYP) [39]. Likewise, B3LYP shows that C2-N3 bond distance in water solvated *n*-pentylcarbonitrile is 1.163 Å longer than the experimental value for the CN bond in acetonitrile (1.157 Å) and hydrogen cyanide (1.156 Å). The B3LYP stretching frequencies for the C-N triple bond and C1-C2 single bonds are 945.9 and 2,338 cm^−1^, respectively, noticeably lower than those in acetonitrile (Table 11). The NBO and Mulliken charge analysis further confirm that *n*-pentyl- carbonitrile and acetonitrile show similar trends; cyano nitrogen with the most negative charge (−0.631) and the cyano carbon is the most positive carbon (0.076) but much less positive compared to the cyano carbon in acetonitrile (0.295). Similar to the acetonitrile model, GIAO ^1^H-NMR simulation (*versus* TMS B3LYP/6-311 + G(2d,p) reference) of *n*-pentylcarbonitrile receptor using the B3LYP optimized structure affords a single peak at 2.54 ppm, slightly more deshielded (2.28 ppm in acetonitrile) for the two protons attached to C1 (Table 11). The cyano carbon (C6) and C1 show similar ^13^C-NMR shifts compared to those in acetonitrile; 107.46 and 9.41 ppm respectively, and slightly more deshielded (103.77 and −8.72 ppm in acetonitrile, respectively).

### 2.7. Magnesium(II) Bound to Two Acetonitrile Receptors

**Figure 7.**
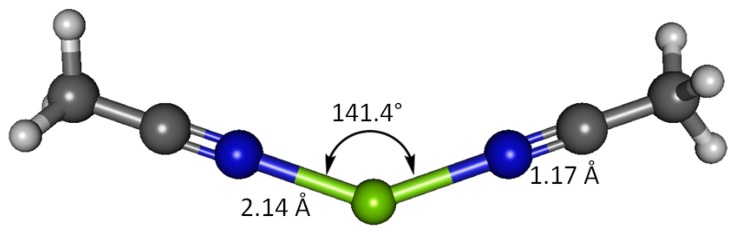
Optimized geometry of Mg(II) complex with two equivalents of acetonitrile.

Interaction between magnesium(II) cation and two acetonitrile receptor molecules in water solvent has been studied using B3LYP and G3 methods (Figure 7). The G3 optimized geometry show that when bound to two acetonitrile molecules, Mg(II) imparts 0.401 of its charge to the receptors, more effectively than it does to one receptor molecule resulting only 80% remains at magnesium (88% in case of one acetonitrile molecule). The nitrogen atom retains more negative charge (−0.176) and the cyano carbon less positive charge (0.356), compared to the binding with only one receptor. The charge on H atoms for both cases is the same (0.124). The imparted positive charge from magnesium in this case is shared equally between two acetonitrile molecules and thus the polarization in the cyano functionality becomes less dramatic. The two receptors bound to Mg(II) form a N-Mg-N angle of 141.4°, in striking contrast to the perfectly linear disubstituted magnesium in species such as MgCl_2_ [49]. The H-C1-H bond angles are close to the ideal tetrahedral angle (109.6°). The differences in the acetonitrile bond lengths in the two cases (1 and 2 equivalents) are essentially negligible—all the C2-N3, C1-C2 and C1-H have essentially the same bond distances. Only the N-Mg bond is longer; 2.142 Å with two equivalents of acetonitrile *versus* 2.131 Å with one equivalent. The C-N and C-C stretching frequencies in the doubly bonded complex are noticeably higher; 969 and 2,623 cm^−1^, respectively, suggesting higher energies required to stretch these bonds. The doubly bound complex affords new bands of interest at 168.2 and 386.8 cm^−1^, attributed to symmetric N-Mg-N bend and asymmetric N-Mg-N stretch, respectively (Table 11).

### 2.8. Malononitrile-Be(II) Complex

**Figure 8.**
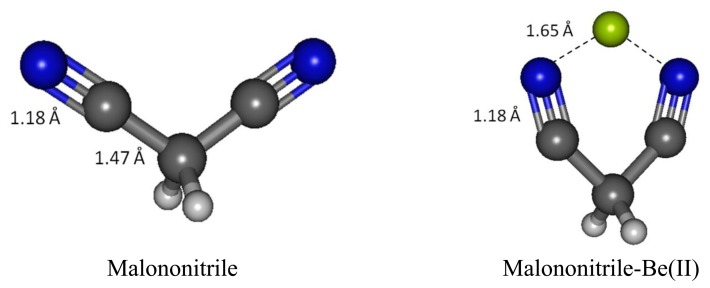
Optimized geometries of malononitrile and its complex with Be(II).

We have examined malononitrile complexes with magnesium and beryllium cations in the gas phase and in water medium. In the aqueous medium of interest only Be(II) complex with one molecule of malonotrile is found to be bound (Figure 8). Magnesium(II) can form a bound complex with only one molecule of malonitrile and the complex remains intact only in the gas phase. On the contrary, beryllium(II) forms complexes with one and two equivalents of malononitrile in vacuum, but only the one equivalent adduct survive in water.

The malononitrile receptor model provides interesting insights on the cage-like complex structure where two carbonitrile functionalities are tied together, separated by only one methylene unit. The two electron lone-pairs can be imagined as pointing at the opposite directions of each other and the two need to come together and bind at the side of the cyano groups with an angle N-Be-N much less than 180° rather than forming a linear adduct as in the acetonitrile-Be(II) described earlier.

**Figure 9.**
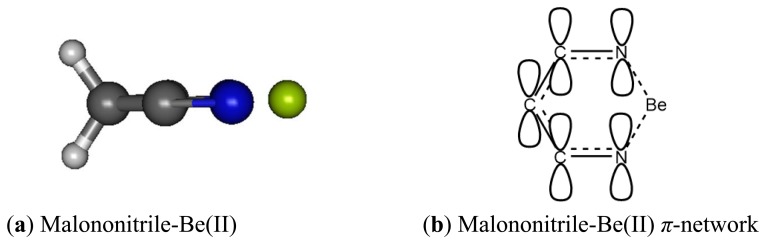
(**a**) Planar optimized structure of Be(II) complex with malononitrile; (**b**) conjugated *π*-network in malononitrile-Be(II).

**Figure 10.**
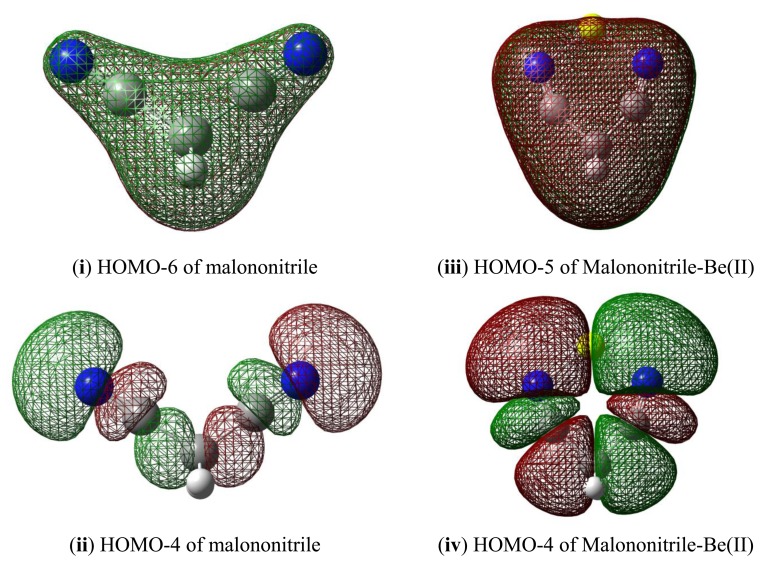
Selected molecular orbitals of malononitrile (**i**) and (**ii**); malononitrile-Be(II) complex (**iii**) and (**iv**).

When binding to malononitrile at both of the cyano groups, Be(II) transfers 0.938 of its charge to the receptor. As a result, the nitrogen becomes more positive by 0.354 and the methylene carbon (C1) has a positive charge built increased by 0.144. The methylene hydrogens also experience an increase in positive charge. In striking contrast to acetonitrile and its complex with Be(II) the cyano carbon in malononitrile and its beryllium complex carry only small positive Mulliken charges. The *σ*-*p* conjugation in malononitrile is presumably very different from the one in acetonitrile described earlier. The methylene carbon does not accumulate negative charge as malononitrile binds to Be(II). The six-membered malononitrile-Be(II) complex is a perfectly flat structure having bond angles of 110.5° for N-Be-N, 106.3° for Be-N-C, 92.6° for C-C-C and 111.3° for H-C-H. The *σ*-*p* conjugated network presumably covers the entire six-membered ring, stabilized by resonance and thus relieves the burden of extreme negative charge on the methylene carbon. In striking contrast to the methyl carbon in acetonitrile, the malononitrile methylene carbon can be considered as having positively charged characteristic wherein the 2*p*_z_ orbital allows conjugation via *π*-network, similar to butadiene or acrolein (Figures 9 and 10).

Beryllium cation binds to both cyano nitrogen atoms in malononitrile, reducing the N-N distance from 4.413 Å to 2.766 Å, putting the Be(II) in the middle between the two nitrogen with 1.761 Å N-Be bond distance. The C-N bond distances in malononitrile and its Be(II) complex are the same (1.179 Å). Likewise, the C1-H bond distances in both cases are also the same (1.094 Å). The C1-C2 bond distance is noticeably longer in the complex (1.483 Å *versus* 1.466 Å).

Malononitrile-Be(II) affords distinct vibrational frequencies at 374.0 cm^−1^ (sym. N-Be-N bending), 578.4 cm^−1^ (antisym. N-Be-N stretch) and 715.3 cm^−1^ (sym. N-Be-N stretch). The symmetric C-C stretch occurs at 977.7 cm^−1^ and C-N stretches occur at 2,517.0 and 2,540.0 cm^−1^ (asymm. and sym. stretches, respectively) (Table 11). ^13^C-NMR GIAO (*versus* TMS B3LYP/6-311 + G(2d,p)) at B3LYP in water medium shows that the cyano carbon and the methylene carbons are both significantly shielded compared to uncomplexed malononitrile; 152.57 and 15.21 ppm, respectively (Table 11). The increase in the cyano carbon shift is very large (56 ppm), presumably attributed to the ring current in the conjugated planar structure of the complex. Likewise, the two ^1^H nuclei bonded to C1 are also apparently deshielded (5.30 ppm, compared to 3.91 ppm in malononitrile). The deshielding of the ^1^H nuclei presumably is similar to the ^1^H nuclei at the periphery and perpendicular to the delocalized network or ring current in aromatic rings such as benzene, furan and imidazole.

### 2.9. Carbonitrile Reactivity and Binding Energies

**Figure 11.**
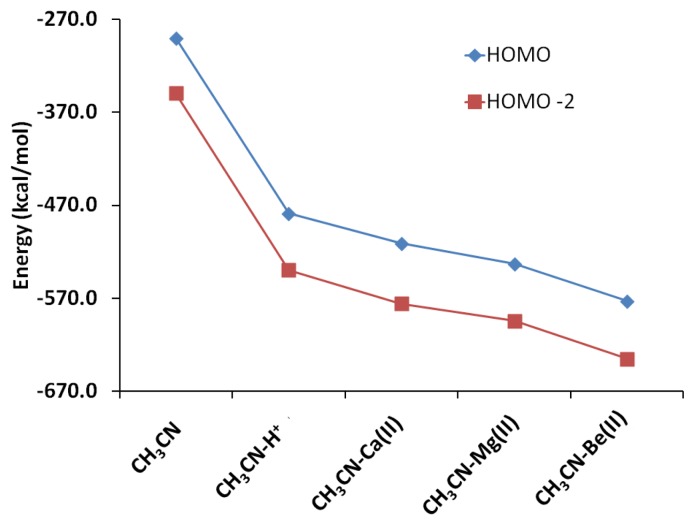
Lowering of acetonitrile MO energies due to interaction with cations [calculated using B3LYP/6-31 + G(d,p)].

The interactions of carbonitriles with cations involve the cyano nitrogen lone-pair electrons, leaving the *π*-electrons alone in order to avoid forming unstable vinyl cation [42–46]. The driving force towards complex formation can be rationalized in terms of stabilization effect or energy lowering on the carbonitrile receptor. As highlighted by Purcell and Drago, interactions of Lewis acids with acetonitrile significantly reduce the energies of the *π*-molecular orbitals in the complexes [50]. This work (Figure 11) confirms this argument. The MO energies of HOMO and HOMO-2 orbitals in the three complexes are much lower than those in acetonitrile. In keeping with the observations discussed earlier (*i.e.*, Figure 5), the HOMO energies follow the general trend of Be(II) > Mg(II) > Ca(II).

### 2.10. Sensor Cell Signals

**Figure 12.**
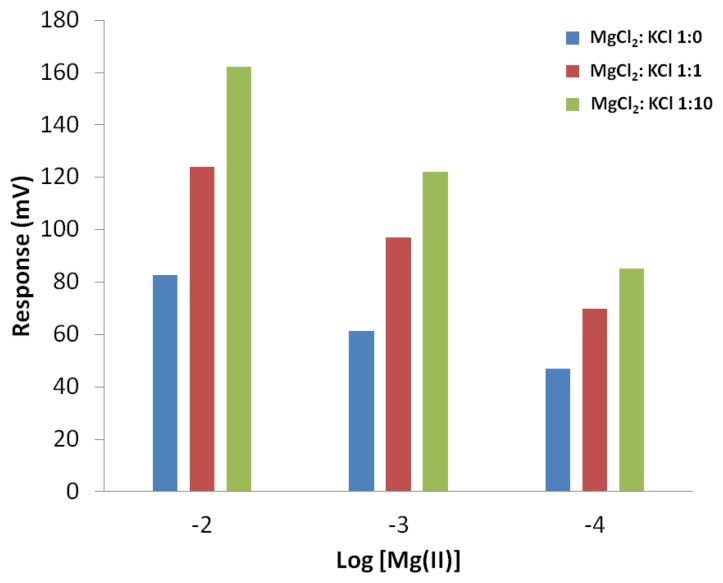
Response of fabricated chemical sensor with cyclopentane carbonitrile immobilized in acrylic copolymer membrane.

Cyclopylcarbonitrile is commercially available and used in this work as the cation recognition agent in a chemical sensor array application. In a potentiometry transduction measurement, target analyte and interfering cations produce voltage signals when the chemical sensor is connected to a reference electrode (typically a double junction silver-silver chloride electrode) [10]. In order to convert analyte concentration to voltage signal and to maintain a stable signal, the sensor is equipped with its own internal reference (such as silver-silver chloride or doped conductive polymer). Potential difference between the internal reference and sensing membrane produces electrical signal according to the Nernst equation [51]. Figure 12 shows the signal patterns of different concentrations of magnesium ion without any interfering ion and with low and high concentrations of potassium ions. The mixture of magnesium-potassium with one to one ratio causes the sensor signal to increase by 40 mV [in comparison to signals from pure Mg(II)] for logarithmic of 1 × 10^−2^ M and 1 × 10^−3^ M but only 20 mV increases for 1 × 10^−4^ M. Similar trend has been observed when the proportion of potassium is increased such that in the magnesium to potassium ratio of 1:10, a signal increase of 40 mV for logarithmic of 1 × 10^−2^ M and an increase of 20 mV for logarithmic of 1 × 10^−3^ and 1 × 10^−4^ M, due to addition of K^+^. With the observed consistent and predictable signal patterns the carbonitrile receptor molecules can be exploited for detection of cations in sensor array applications.

### 2.11. IR Measurement

**Figure 13.**
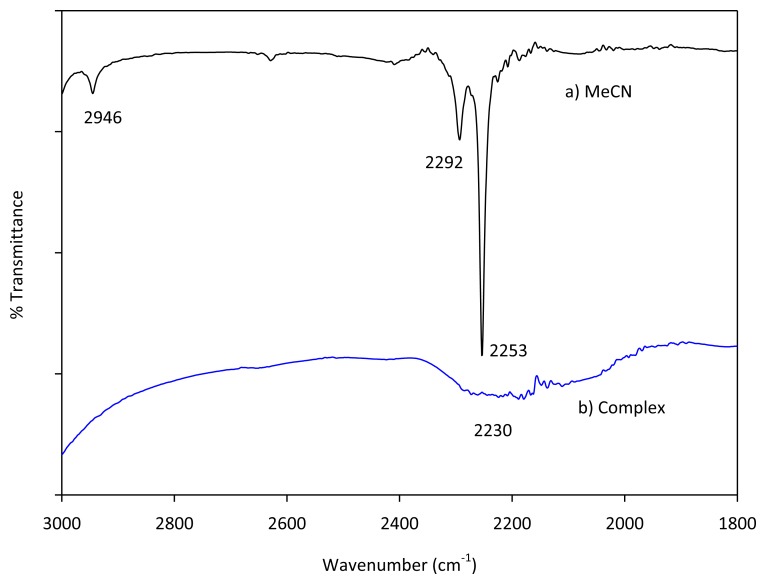
The IR spectra of acetonitrile and acetonitrile-Mg(II) complex.

As described earlier our simulation using the G3 method reveals that uncomplexed acetonitrile shows a vibrational frequency at 2,586.1 cm^−1^ that can be attributed to C-N stretching. Likewise, as expected based on earlier experimental results, binding with cations increase the frequencies of the cyano group; Mg(II), Be(II) and H^+^ afford the following C-N stretches 2,620.8, 2,623.0 and 2,609.0 cm^−1^, respectively. Purcell and Drago have reported facile preparations of acetonitrile complexes with boron trifluoride and tin tetrachloride [50]. However, when we tried to prepare magnesium complex with acetonitrile using the method described in the next section, we only observed only broad peak at the 2,300 to 2,000 cm^−1^ region (Figure 13). However, using the same characterization setup, neat acetonitrile affords a strong cyano C-N stretching frequency at 2,253 cm^−1^.

## 3. Experimental Section

### 3.1. Computational Details

Calculations were performed with a Gaussian 09 Revision C.01 program package [52]. All calculations were initially performed with B3LYP using the split valence basis set with polarization and diffuse functions for heavy atoms. All geometry optimization and electronic energies are initially obtained at the B3LYP/6-31 + G(d,p) level [53,54]. Gaussian NBO version 3.1 has been utilized to calculate atomic orbital occupancies and its contribution to bonding interaction and delocalization of electron density within the receptor models and its complexes [41]. The ^13^C-NMR isotropic shielding was calculated with the GIAO method using the optimized structures obtained from DFT calculation [55]. The IEF version of the PCM solvation method was employed to estimate the effect of water solvation on binding energies [28].

The G3 and CBS-QB3 method were employed in four benchmark cases and throughout this work. G3 geometry optimization [31,32] is done at MP2 level using smaller 6-31G basis instead of the 6-311G basis employed in G2 Theory [56], and the structure is used to calculate harmonic frequencies and for all subsequent calculations. The final MP2 calculations use a larger basis (G3large) and both the core and valence electrons are correlated. Correction for correlation effects beyond a fourth-order pertubation theory is done with quadratic configuration interaction calculation with single and double excitations and a triples excitation contribution [QCISD(T)] [30]. Additional core correlation corrections are included through a spin-orbit and valence electron empirical correction. Different empirical parameters are also introduced in the higher level correction (HLC) [57].

Instead of using additive correction to account for the limitations of the basis sets, the key in CBS methods such as the quadratic CI-based CBS-Q and CBS-QB3, is the extrapolation of the basis set to an infinite limit [58]. CBS-QB3 combines the general CBS-Q design with B3LYP DFT optimized geometry and frequencies along with single point calculations at CCSD(T), MP4SDQ and MP2 levels. G4 Theory uses B3LYP/6-31G(2df,p) instead of MP2(full)/6-31G* (in G3), for structure optimization, harmonic frequency and all subsequent calculations [33]. Hartree-Fock energy limit E (HF/limit) is determined using two-point extrapolation scheme and Dunning's aug-cc-pVnZ basis sets. For correlation correction beyond the fourth-order pertubation theory, CCSD(T)/6-31G* is used instead of QCISD(T)/6-31G* (in G3). The HLC has the same form as in G3 but with two additional parameters. MQZVP basis sets are parts of Truhlar's M06 and M06-2X functional [59,60]. These are modified version of Ahlrich's higher polarization QZVP basis set recommended for correlated treatments [61,62]. The Ga to Kr QZVP bases include a diffuse *d* function to polarize 4*p* AO and add 2*f*1*g* sets from Dunning [63].

### 3.2. Sensor Fabrication

Methyl methacrylate (MMA), *n*-butyl acrylate (nBA), 2-hydroxyethyl methacrylate (HEMA) hexanediol diacrylate (HDDA) crosslinker, 2,2-dimethoxy-2-phenylacetophenone (DMPP) photoinitiator and sodium tetraphenyl borate lipophilic agent were purchased from Sigma-Aldrich (Milwaukee, WI, USA). Cyclopentane carbonitrile cation recognition molecule was procured from Fluka (Buchs, Switzerland). Magnesium chloride and potassium chloride were obtained from Merck KGaA (Darmstadt, Germany). All chemicals were used as received without further purification.

The sensing membrane is a self-plasticized acrylic co-polymer having 2 parts of MMA and 8 parts of n-BA, by volume, called MB28. Initially, 100 μL of MMA, 400 μL of nBA, 6.2 mg of DMPP and 0.5 μL HDDA were prepared as MB28 monomer mixture. Later, 100 μL of the mixture was mixed with 1 mg of sodium tetraphenylborate and 5 μL of cyclopentylcarbonitrile to afford the cation sensing cocktail.

Poly(HEMA) was used as the internal layer that provides a constant concentration of cation. The HEMA cocktail was prepared by mixing 500 μL of HEMA, 6.2 mg of DMPP and 0.5 μL HDDA. The electrochemical transducer electrode was prepared by screen printing of silver-silver chloride paste onto a FR4 printed board and oven dried at 120 °C to give a dry thickness of 100 μm. The HEMA cocktail (2 μL) was dispensed on the silver-silver chloride electrode and photocured under UV for 3 min. The photocured poly(HEMA) hydrogel was hydrated by conditioning in 0.1 M KCl for 10 min. The MB28 cocktail (3 μL) was dispensed on top of the hydrated poly(HEMA) hydrogel and photocured under UV for 3 min. Three concentrations (1 × 10^−4^, 1 × 10^−3^ and 1 × 10^−2^ M) of Mg(II) and K^+^ were prepared by dissolving the chloride salts in deionized water. Mg(II) and K^+^ mixtures in 1:1 and 1:10 were also prepared in order to investigate the sensor response in mixtures. The sensing electrode and a double-junction reference electrode (Thermo Fisher Scientific, Waltham, MA, USA) are connected to Thermo Orion ion meter, immersed in the calibration solutions and the potential signals recorded.

### 3.3. IR Measurement

The cyano C-N stretching frequencies of neat acetonitrile and acetonitrile-Mg(II) complex were characterized using a Perkin-Elmer ATR instrument. The acetonitrile-Mg(II) complex is prepared as follows; magnesium chloride is dissolved in acetonitrile assisted with minimal amount of water. The mixture is stirred overnight, the excess liquid filtered off and the residue was exposed to mild heat treatment in the oven to achieve complete dryness. A few dry crystals (neat) are compacted on diamond plate for IR measurement.

## 4. Conclusions

In this paper we have presented a systematic theoretical study of cation recognition using carbonitriles as receptors for the design of chemical sensor array. We have employed B3LYP, G3, CBS-QB3, G4 and MQZVP methods in four benchmark cases relevant to chemical sensor design. The methods had been verified as accurate and diligently used for designing cation recognition molecules. The G3 method has been especially useful due to its accuracy and speed in evaluating the binding interactions between carbonitriles and Mg(II), Be(II) and H^+^. The G4 method has been specifically adopted to gain accurate energy calculations involving calcium(II). The G4 reproduces the earlier G3 results of Mg(II), Be(II) and H^+^. ^1^H-NMR study shows that H6 is deshielded by interaction with cations following the trend Be(II) > Mg(II) > Ca(II). The results also show that H6 can be distinguished from H4 and H5 in the acetonitrile-analyte complexes, presumably due to σ-*p* conjugation. The hydrogen atoms on C1 and N3 receive most of the positive charge from the analytes. NBO analysis shows that the cyano triple bond remains unchanged after interacting with the analytes.

The *n*-pentylcarbonitrile lipophilic model shows no significant difference from acetonitrile when bound to Mg(II). The complex of Mg(II) with two acetonitrile molecules shows interesting 141.4° N-Mg(II)-N angle in striking contrast to linear MgCl_2_. Malononitrile-Be(II) is a planar complex exhibiting a π-network with cationic methylene carbons as evidenced by its strongly deshielded methylene protons. Frontier orbital analysis shows that interaction with cation lowers the energy of HOMO and HOMO-2 of the carbonitrile receptors. Reproducible sensor signals in Mg(II) separate solutions, and in mixtures of Mg(II) and K^+^ strongly suggest that carbonitrile recognition molecules can be exploited in sensor array applications.

## Supplementary Material



## Figures and Tables

**Table 1.** Bond distances (*r*) and dissociation energies (D_e_) of water dimer benchmark [23].

**2(H**_2_**O)**	**G3**	**CBS-QB3**	**MQZVP**	**Exptl[a]**
H-O (*r*_H-O_, Å)	1.954	1.925	1.956	NA
O-O (*r*_O-O_, Å)	2.913	2.886	2.918	2.978
H-O Freq (cm^−1^)	181.3	204.0	184.6	186.83
D_e_ (kcal/mol)	3.5	3.4	3.1	3.16

[a] Experimental values from references. [24,25].

**Table 2.** Bond distances (*r*) and dissociation energies (D_e_) of ammonia-borane benchmark.

**NH_3_-BH_3_**	**G3 Vac**	**CBS-QB3 Vac**	**MQZVP Vac**	**G3 Water[b]**	**CBS-QB3 Water[b]**	**MQZVP Water[b]**		**Exptl[a]**
B-N (*r*_B-N_, Å)	1.661	1.664	1.658	1.626	1.627	1.630	1.580	
B-H (*r*_B-H_, Å)	1.209	1.208	1.206	1.214	1.213	1.190	1.150	
N-H (*r*_N-H_, Å)	1.020	1.017	1.014	1.020	1.018	1.010	0.960	
B-N Freq (cm^−1^)	605.0	636.5	642.4	NA	722.4	NA	NA	
D_e_ (kcal/mol)	28.0	27.8	24.8	35.1	34.9	31.8	NA	

[a] Experimental values from references. [26,27].

[b] Water solvation effect is modeled using iefpcm method as in reference [28].

**Table 3.** G3 and G4 geometries and binding energies of Mg(II) complexes with HCN and H_2_O †.

**Mg(II)-NCH**	**G3[a]**	**G4[b]**	**Mg(II)-OH_2_**	**G3[a]**	**G4[b]**
Mg-N (*r*_Mg-N_, Å)	2.016	1.965	Mg-O (*r*_Mg-O_, Å)	1.948	1.917
N-C (*r*_N-C_, Å)	1.168	1.149	O-H (*r*_O-H_, Å)	0.989	0.979
Mg-N Freq (cm^−1^)	498.7	510.2	Mg-O Freq (cm^−1^)	563.1	571.5
D_e_ (kcal/mol)	92.0	92.6	D_e_ (kcal/mol)	80.6	81.5

[a] The G3 method refer to references. [30–32].

[b] The G4 method refer to reference [33].

† Experimental data are unavailable.

**Table 4.** Bond distances of acetonitrile and its complexes with Be(II), Mg(II) and H^+^.

**Molecule**[a]	**Bond Distance (Å)**[b]				**Bond Angle**[b]		
	
	**C1-C2**	**C2-N3**	**N3-M**	**C1-H**	**C2-N3-M**	**H6-C1-C2**	**H5-C1-H4**
CH_3_CN-Be(II)	1.444	1.165	1.625	1.092	175.5	108.6	110.1
CH_3_CN-Mg(II)	1.453	1.170	2.131	1.091	172.5	109.6	109.6
CH_3_CN-H^+^	1.445	1.155	1.014	1.091	180.0	108.7	110.2
CH_3_CN	1.460	1.178	NA	1.091	NA	109.9	109.0

[a] Numbering of atoms according to Scheme 1.

[b] Bond distances and bond angles obtained from G3 calculations.

**Table 5.** ^1^H- and ^13^C-NMR shifts (ppm) of acetonitrile and its complexes with Be(II), Mg(II) and H^+^.

**Molecule**[a]	**C1**	**C2**	**N3**	**H6**	**H4,H5**
CH_3_CN-Be(II)	−8.37	115.36	155.96	3.16	3.13
CH_3_CN-Mg(II)	−8.18	111.03	187.74	2.64	2.67
CH_3_CN-H^+^	−9.93	86.50	186.39	2.94	2.94
CH_3_CN	−8.72	103.77	232.75	2.28	2.28

[a] Numbering of atoms according to Scheme 1.

[b] Bond distances and bond angles obtained from B3LYP/6-31 + G(d,p) calculations.

**Table 6.** Mulliken charge [a] of acetonitrile and its complexes with Be(II), Mg(II) and H^+^.

**Molecule**[b]	**C1**	**C2**	**N3**	**M**	**H6**	**H4,H5**
CH_3_CN-Be(II)	−0.239	0.364	0.212	1.252	0.136	0.137
CH_3_CN-Mg(II)	−0.362	0.379	−0.141	1.754	0.124	0.123
CH_3_CN-H^+^	−0.082	0.392	−0.095	0.368	0.139	0.139
CH_3_CN	−0.097	0.295	−0.516	NA	0.106	0.106

[a] Mulliken charge obtained from G3 calculations.

[b] Numbering of atoms according to Scheme 1.

**Table 7.** Vibrational stretching frequencies (cm^−1^) of acetonitrile and its Be(II), Mg(II) and H^+^ complexes.

**Molecule**[a]	**C1-C2**	**C2-N3**	**N3-M**
CH_3_CN-Be(II)	1,056.8	2,623.0	728.2
CH_3_CN-Mg(II)	966.7	2,620.8	311.4
CH_3_CN-H^+^	920.6	2,609.0	3,938.0
CH_3_CN [b,c]	954.8	2,586.1	NA

[a] Numbering of atoms according to Scheme 1.

[b] Vibrational frequencies of acetonitrile were obtained using RHF calculations and the rest using the G3 method.

[c] The vibrational frequencies of ethane calculated at B3LYP/6-31 + G(d,p) for comparison purposes.

**Table 8.** † Orbital occupancies of acetonitrile and its complexes with Be(II), Mg(II) and H^+^.

	**2*s***	**2*P*_x_**	**2*P*_y_**	**2*P*_z_**	**Total**
**CH**_3_**CN-Mg(II)**					
**C2**	0.875	0.974	0.793	0.793	3.435
**N3**	1.542	1.583	1.244	1.244	5.614
**C1**	1.149	1.056	1.310	1.310	4.824
**CH**_3_**CN-Be(II)**					
**C2**	0.876	0.962	0.726	0.727	3.291
**N3**	1.497	1.607	1.324	1.323	5.751
**C1**	1.155	1.043	1.316	1.317	4.830
**CH**_3_**CN-H**^+^					
**C2**	0.866	0.957	0.734	0.734	3.291
**N3**	1.298	1.467	1.323	1.323	5.411
**C1**	1.156	1.035	1.320	1.320	4.830
**CH**_3_**CN**					
**C2**	0.887	0.987	0.879	0.879	3.631
**N3**	1.598	1.511	1.147	1.147	5.403
**C1**	1.138	1.076	1.297	1.297	4.808

† Calculated using B3LYP/6-31 + G(d,p).

**Table 9.** *s versus p* characters in C2-N3 and C1-C2 *σ*-bonds in acetonitrile and its Mg(II), Be(II) and H complexes †.

**C2-N3 σ-bond *s*and *p* characters by percentage**				
	**C2% *s***	**C2% *p***	**N3% *s***	**N3% *p***
CH_3_CN-Be(II)	44.8	55.2	53.2	46.5
CH_3_CN-Mg(II)	43.4	56.5	57.9	42.0
CH_3_CN-H^+^	43.6	56.3	51.9	48.1
CH_3_CN	46.5	53.5	45.1	54.5

**C1-C2 σ-bond *s*and *p* characters by percentage**				
	**C1 % *s***	**C1 % *p***	**C2 % *s***	**C2 % *p***

CH_3_CN-Be(II)	23.6	76.4	55.4	44.6
CH_3_CN-Mg(II)	22.9	77.0	56.6	43.4
CH_3_CN-H^+^	56.6	43.4	22.7	77.2
CH_3_CN	24.2	75.7	53.8	46.2

† Calculated using B3LYP/6-31 + G(d,p).

**Table 10.** G3, CBS-QB3 and G4 binding energies of acetonitrile complexes with Mg(II), Be(II) and H^+^ in vacuum and water †.

**Complex**	**G3 ΔH_rxn_(kcal/mol)**		**CBS-QB3ΔH_rxn_(kcal/mol)**		**G4 ΔH_rxn_(kcal/mol)**
		
	**Vac**	**H_2_O**	**Vac**	**H_2_O**	**Vac**
CH_3_CN-Be(II)→Be(II) + CH_3_CN	187.2	42.3	185.1	40.7	187.4
CH_3_CN-Mg(II)→Mg(II) + CH_3_CN	112.4	12.5	109.1	10.4	112.8
CH_3_CN-H^+^→H^+^ + CH_3_CN	NA	186.7	NA	138.2	186.8
CH_3_CN-Ca(II)→Ca(II) + CH_3_CN	NA	NA	NA	NA	79.5

† Binding energies were calculated using G3 and CBS-QB3 methods. Water solvation effect was accounted for using iefpcm model.

**Table 11.** Vibrational frequencies and NMR shifts of Mg(II) and Be(II) complexes with *n*-pentylcarbonitrile, two equivalents of acetonitrile, malononitrile and the uncomplexed receptors.

**Molecule**	**Vibrational Frequency (cm**^−1^**)**				**NMR Shift (ppm)**		
	
	**C-N**	**C-C**[a]	**N-Mg**	**N-Be**	**C1**	**C_cyano_**	**H_(on C1)_**
*n*-Pentylcarbonitrile [c]	2,338.4	945.9	NA	NA	9.41	107.46	2.54
Malononitrile [d]	2,631.8	1,044.3	NA	NA	−0.05	96.36	3.91
*n*-Pentylcarbonitrile [c]- Mg(II)	2,381.5	965.2	413.3	NA	8.46	111.51	2.92
(Acetonitrile)_2_[d]- Mg(II)	2,623.5	969.0	386.6	NA	−8.32	110.25	2.62
Malononitrile [d]-Be(II)	2,540.0	977.7	NA	NA [b]	15.21	152.57	5.30

[a] C-C refers to methylene carbon atoms.

[b] No value for the corresponding stretch.

[c] The geometry was optimized at B3LYP/6-31 + G(d,p) with iefpcm solvation model.

[d] The geometry was optimized using G3 with iefpcm solvation model.
