# Supplementary material for: G3 Assisted Rational Design of Chemical Sensor Array Using Carbonitrile Neutral Receptors
Source: Sensors (Basel). 2013 Oct 14;13(10):13835–60. doi: 10.3390/s131013835 (PMC3859094; doi:10.3390/s131013835)
Supplement: Supplementary file 1 [file sensors-13-13835-s001.pdf]

OPEN ACCESS
sensors
ISSN 1424-8220
www.mdpi.com/journal/sensors

Supplementary Information

Rational Design of Chemical Sensor Array Using Carbonitrile Neutral Receptors. *Sensors* 2013, *13*, 13835-13860

Ahmad Nazmi Rosli [1,*], Maizathul Akmam Abu Bakar [1,†], Ninie Suhana Abdul Manan [1,†], Pei Meng Woi [1,†], Vannajan Sanghiran Lee [1,†], Sharifuddin Md Zain [1,†], Mohd Rais Ahmad [2,†] and Yatimah Alias [1,†]

[1] Department of Chemistry, Faculty of Science Building, University of Malaya, Kuala Lumpur 50603, Malaysia; E-Mails: maizathul_akmam@yahoo.com (M.A.A.B.); niniemanan@um.edu.my (N.S.A.M.); pmwoi@um.edu.my (P.M.W.); vannajan@gmail.com (V.S.L.); smzain@um.edu.my (S.M.Z.); yatimah70@um.edu.my (Y.A.)

[2] NEMS & Photonics Laboratory, MIMOS Berhad, Technology Park Malaysia, Kuala Lumpur 57000, Malaysia; E-Mail: mrahmad@mimos.my

† These authors contributed equally to this work.

* Author to whom correspondence should be addressed; E-Mail: nazmi86@siswa.um.edu.my; Tel.: +60-3-7967-6774.

---

1. Benchmark Structures

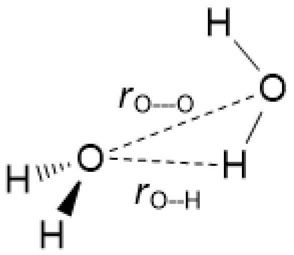

i. water dimer

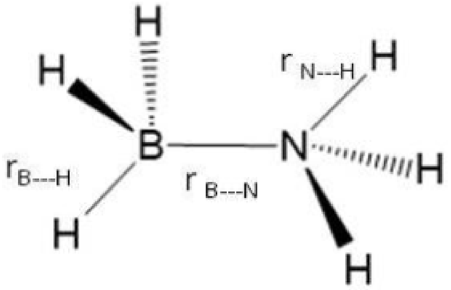

ii. ammonia-borane

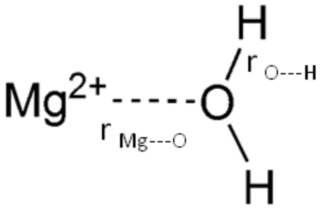

iii. Mg(II)-H2O

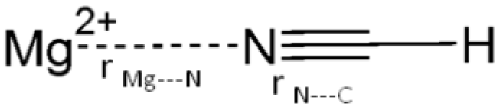

iv. Mg(II)-HCN

HCN

Symbol	*X*	*Y*	*Z*
C	*0*	*0*	*−0.5117*
N	*0*	*0*	*0.664423*
H	*0*	*0*	*−1.58079*

Mg(II)-H_2O

Symbol	*X*	*Y*	*Z*
O	*0*	*0*	*−1.00818*
H	*0*	*0.786319*	*−1.60806*
H	*0*	*−0.78632*	*−1.60806*
Mg	*0*	*0*	*0.940131*

Mg(II)-HCN

Symbol	*X*	*Y*	*Z*
Mg	*0*	*0*	*1.441744*
N	*0*	*0*	*−0.57422*
C	*0*	*0*	*−1.74209*
H	*0*	*0*	*−2.82887*

NH_3—BH_3

**Symbol**	***X***	***Y***	***Z***
**N**	**−0.71562**	**0.000025**	**0.000009**
**B**	**0.910729**	**−1.2E-05**	**−9E-06**
**H**	**−1.09918**	**−0.27609**	**−0.90494**
**H**	**−1.09925**	**0.921743**	**0.213351**
**H**	**−1.09923**	**−0.6456**	**0.691599**
**H**	**1.25129**	**0.341064**	**1.114228**
**H**	**1.250837**	**−1.13564**	**−0.26173**
**H**	**1.251215**	**0.794417**	**−0.85252**

2. Geometrical Structures

CH_3CN

**Symbol**	***X***	***Y***	***Z***
**C**	**0.273053**	**0.000036**	**0.000074**
**N**	**1.450787**	**−1.4E-05**	**−3.1E-05**
**C**	**−1.18659**	**−5E-06**	**−8E-06**
**H**	**−1.55806**	**0.578153**	**−0.84747**
**H**	**−1.55804**	**−1.023**	**−0.07707**
**H**	**−1.55821**	**0.44476**	**0.92436**

Sum of electronic and thermal Enthalpies
= −131.883190 hartrees

Mg—CH_3CN

Symbol	*X*	*Y*	*Z*
C	−0.98716	−0.04353	0.00006
N	0.1805	−0.11206	−1.8E-05
C	−2.43712	0.051242	−8E-06
Mg	2.306119	0.042253	−3E-06
H	−2.72551	1.103334	−0.01879
H	−2.83394	−0.45229	−0.88248
H	−2.83178	−0.41993	0.901111

Sum of electronic and thermal Enthalpies
= −331.340853 hartrees

Be—CH_3CN

Symbol	*X*	*Y*	*Z*
C	0.202038	−0.02106	0.00021
N	−0.96325	−0.04274	0.000144
C	1.645698	0.018501	−9.6E-05
H	2.012613	−0.395	0.941093
H	2.017835	−0.57094	−0.83993
H	1.965726	1.057331	−0.10184
Be	−2.58497	0.055774	−0.00025

Sum of electronic and thermal Enthalpies
= −146.253335 hartrees

Malono nitrile

Symbol	*X*	*Y*	*Z*
C	−1.20945	0.035464	0.000048
C	0	0.86391	0.000049
C	1.209446	0.035465	−4.3E-05
H	−3.2E-05	1.510394	−0.88341
H	0.000034	1.510297	0.883583
N	2.190386	−0.61641	−0.00012
N	−2.19039	−0.61641	0.000047

Sum of electronic and thermal Enthalpies
= −223.604231 hartees

Be—Malono nitrile

Symbol	*X*	*Y*	*Z*
C	1.072616	−0.38718	0
C	0	−1.4118	0.000003
C	−1.07262	−0.38718	0.000001
H	−1E-06	−2.02876	−0.90343
Be	0	1.735182	−2E-06
N	−1.44656	0.730978	−1E-06
N	1.446562	0.730977	−2E-06
H	0	−2.02876	0.903441

Sum of electronic and thermal Enthalpies
= −237.904143 hartees

n-Pentane carbonitrile

Symbol	*X*	*Y*	*Z*
C	−1.51004	0.698612	0.000025
C	−0.34329	−0.31358	−2E-06
C	1.018701	0.390409	−3E-06
C	2.198832	−0.59019	0.000031
C	3.562087	0.11097	−3.3E-05
C	−2.82349	0.051256	−0.00001
N	−3.85925	−0.47726	−2.4E-05
H	−1.45642	1.348367	0.881268
H	−1.45639	1.348435	−0.88117
H	−0.4295	-0.95983	0.881359
H	−0.42952	-0.95981	−0.88137
H	1.089979	1.044742	0.880264
H	1.090016	1.044713	−0.88029
H	2.124338	−1.24476	0.879088
H	2.124294	−1.24496	−0.87887
H	3.680138	0.746221	0.885851
H	3.679016	0.748205	−0.88465
H	4.381984	−0.61541	−0.00137

Sum of electronic and thermal Enthalpies

= −289.875391 hartrees

Mg--n-Pentane carbonitrile

Symbol	*X*	*Y*	*Z*
C	−0.35078	1.051676	−0.00016
C	0.661714	−0.12041	0.000039
C	2.106823	0.391662	0.000031
C	3.133631	−0.74925	−0.00014
C	4.582535	−0.2488	0.000091
C	−1.7287	0.585066	0.000074
N	−2.81294	0.181437	0.000177
H	−0.22021	1.687538	0.883119
H	−0.22029	1.687097	−0.88376
H	0.48477	−0.7446	0.882675
H	0.484807	−0.74484	−0.88244
H	2.268574	1.02775	0.881041
H	2.268527	1.027944	−0.88085
H	2.965515	−1.38574	0.878959
H	2.965642	−1.38533	−0.87955
H	4.788885	0.363893	0.885586
H	4.789054	0.36424	−0.88512
H	5.290031	−1.08485	−9E-06
Mg	−4.71717	−0.62857	−4.3E-05

Sum of electronic and thermal Enthalpies

= −489.750237 hartrees

3. IR spectra

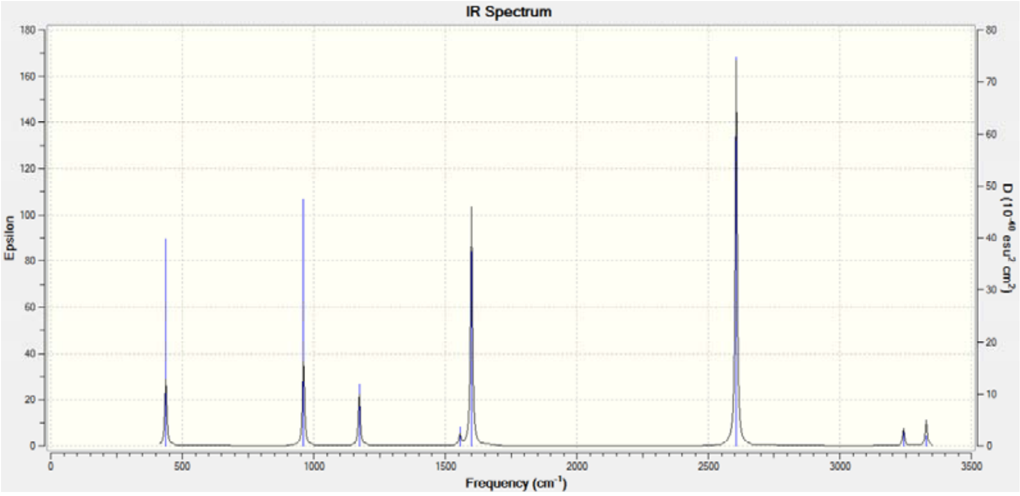

CH_3CN

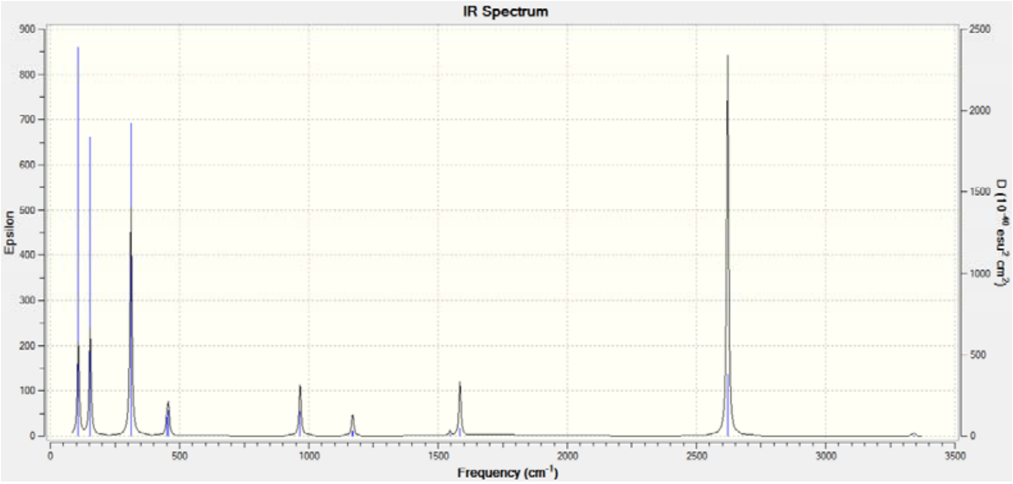

Mg—CH_3CN

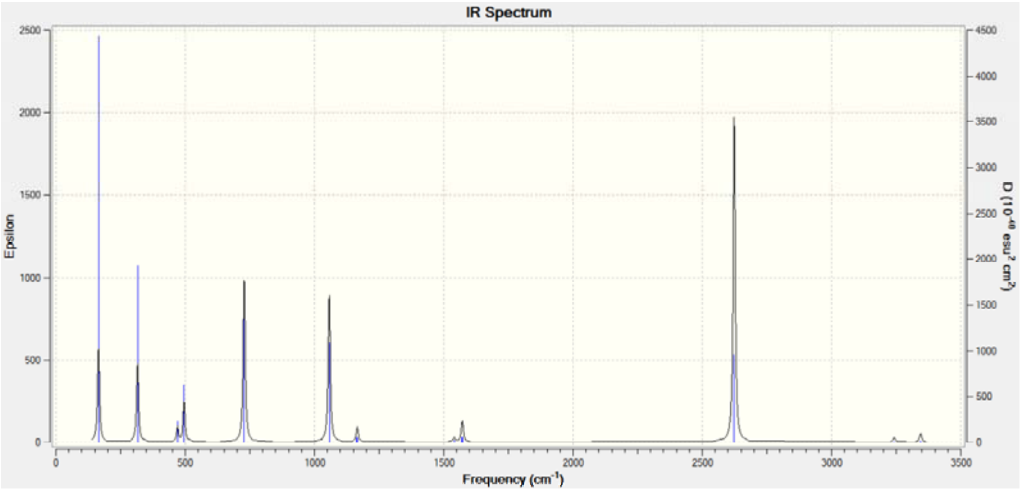

Be—CH_3CN

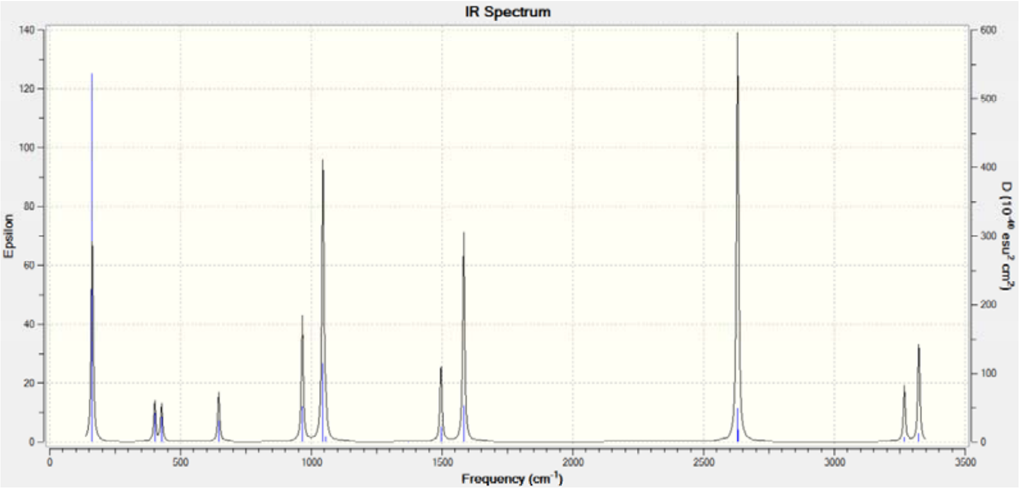

Malono Nitrile

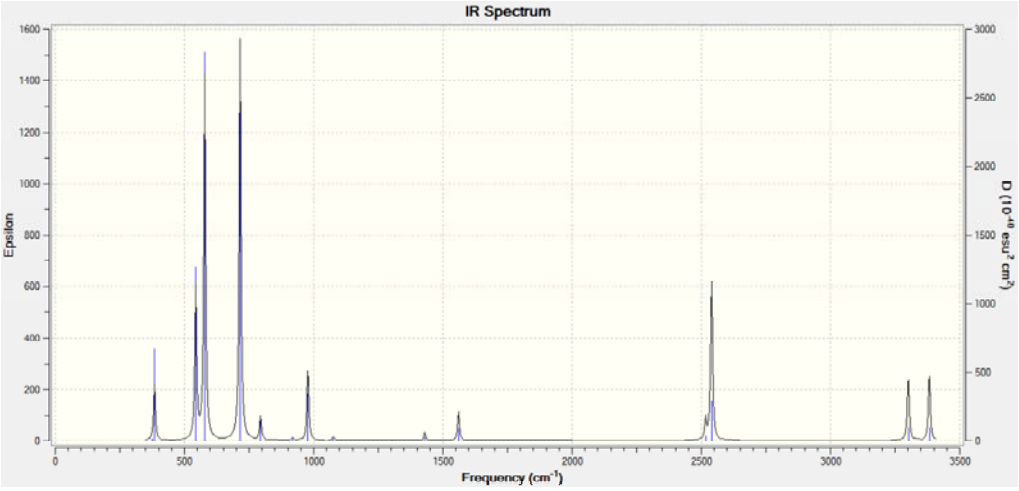

Be—Malono Nitrile

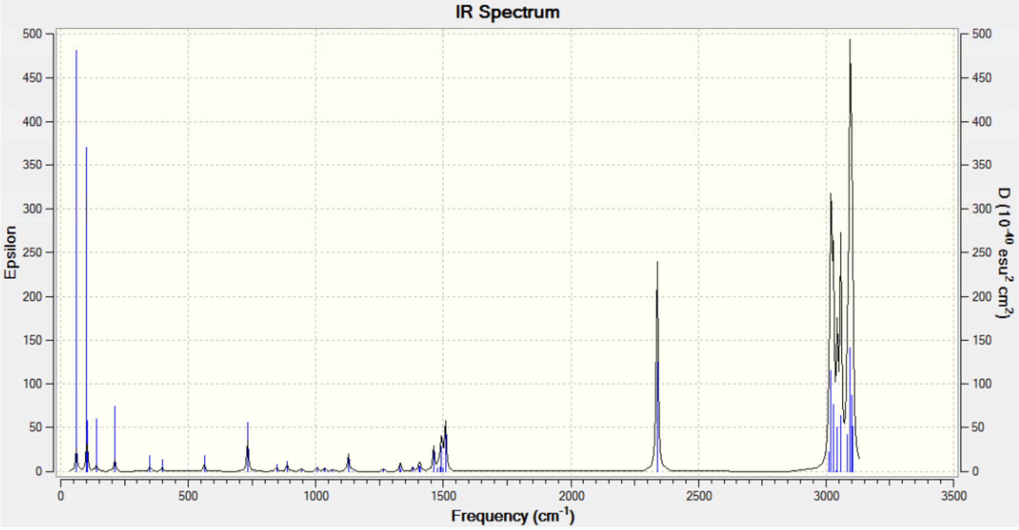

n-Pentane Carbonitrile

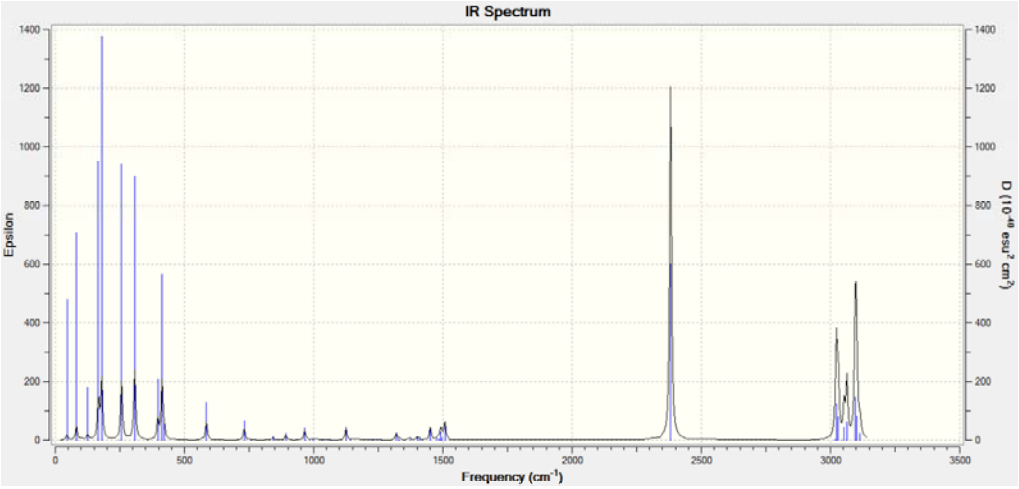

Mg--*n*-Pentane Carbonitrile

4. NMR GIAO Spectra

CH3CN

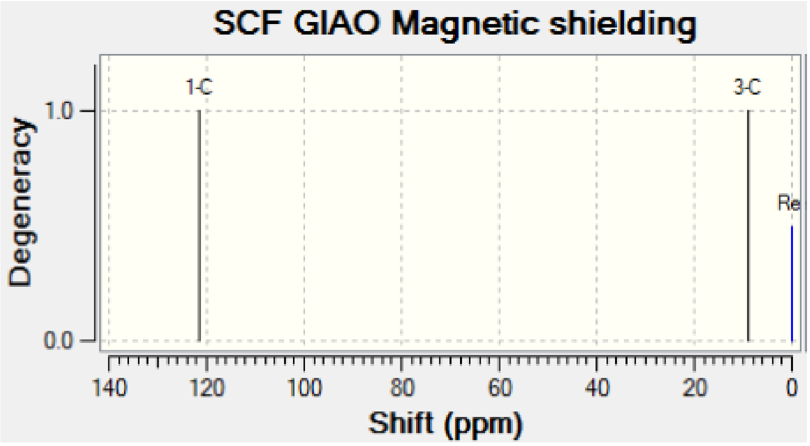

^{13}C NMR GIAO against reference TMS HF/6-31G(d) GIAO

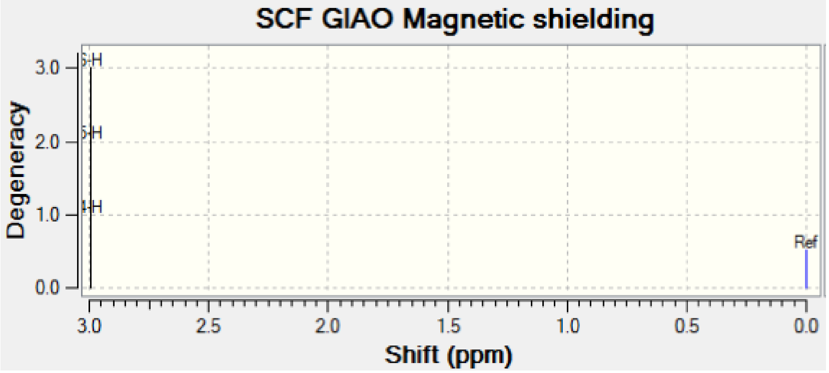

^{1}H NMR GIAO against reference TMS HF/6-31G(d) GIAO

Mg—CH3CN

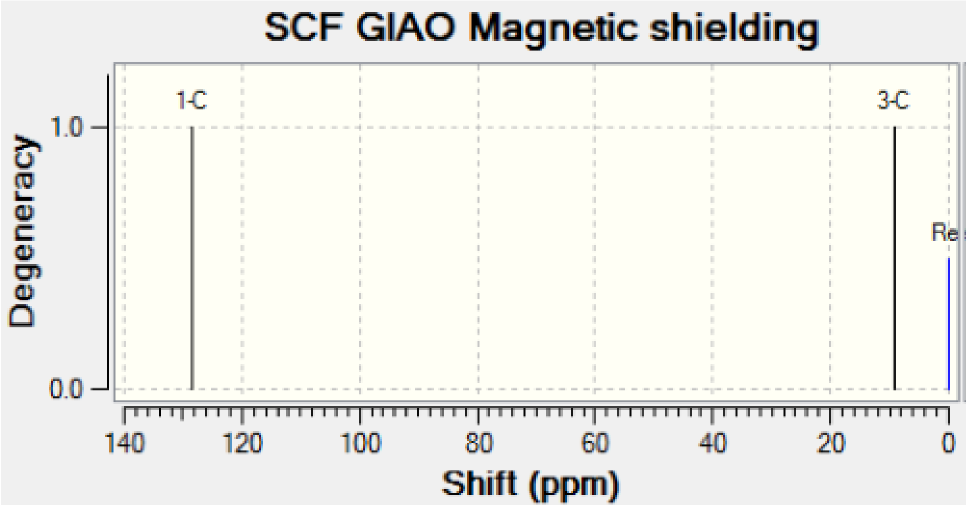

^{13}C NMR GIAO against reference TMS HF/6-31G(d) GIAO

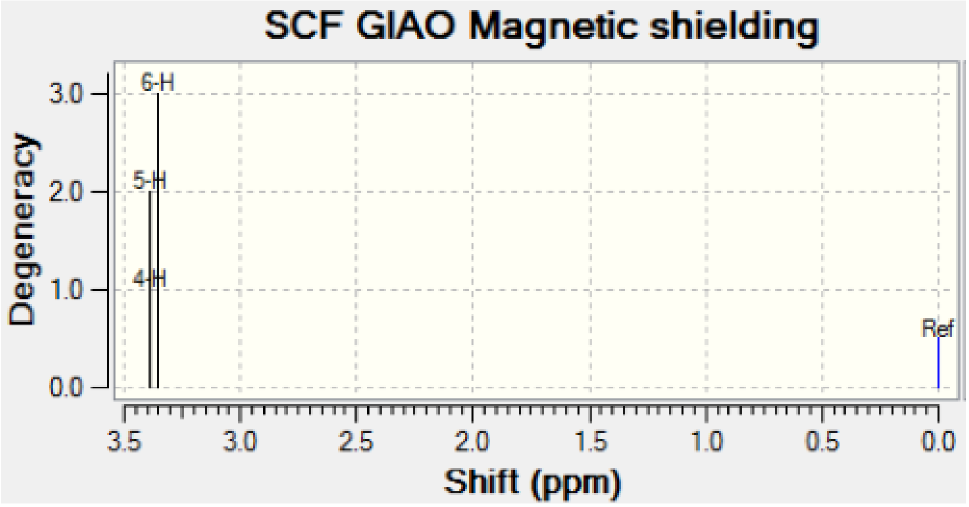

^{1}H NMR GIAO against reference TMS HF/6-31G(d) GIAO

n-Pentane Carbonitrile

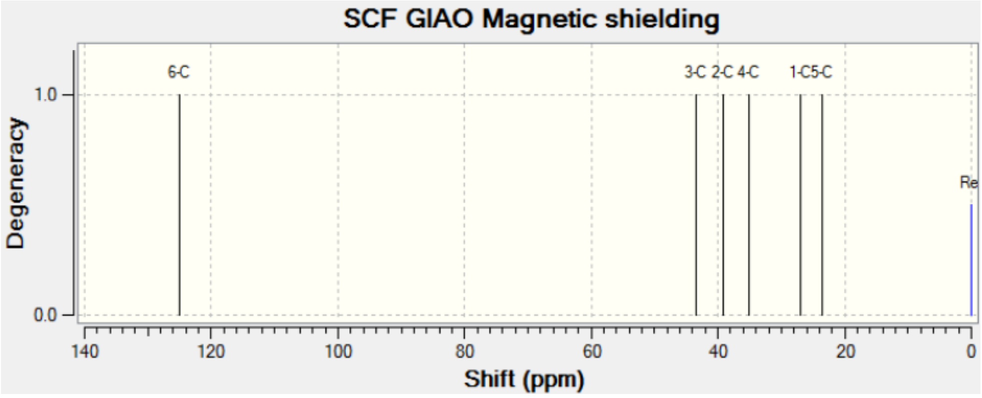

^{13}C NMR GIAO against reference TMS HF/6-31G(d) GIAO

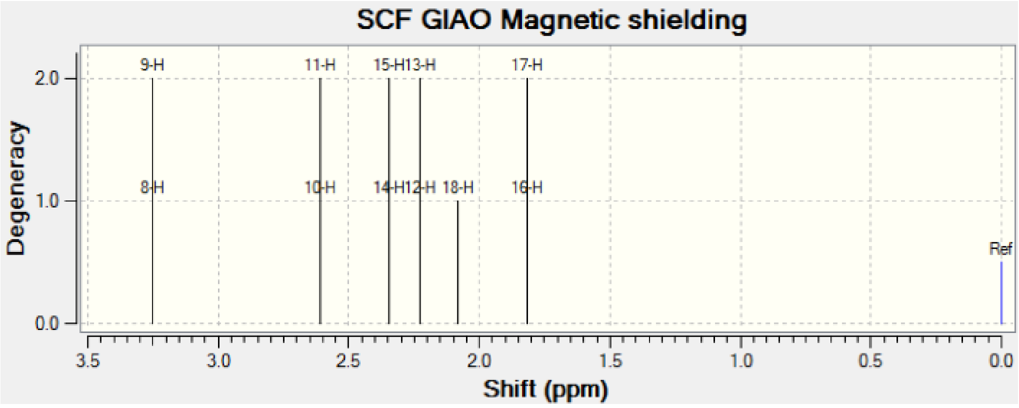

^{1}H NMR GIAO against reference TMS HF/6-31G(d) GIAO

Mg--*n*-Pentane Carbonitrile

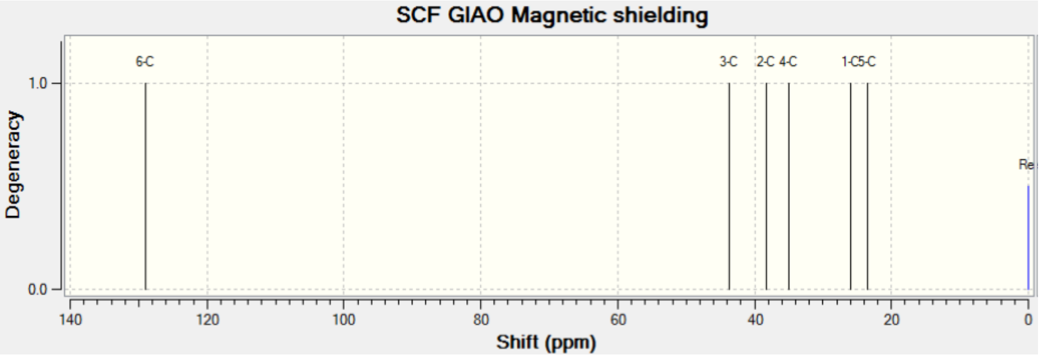

^{13}C NMR GIAO against reference TMS HF/6-31G(d) GIAO

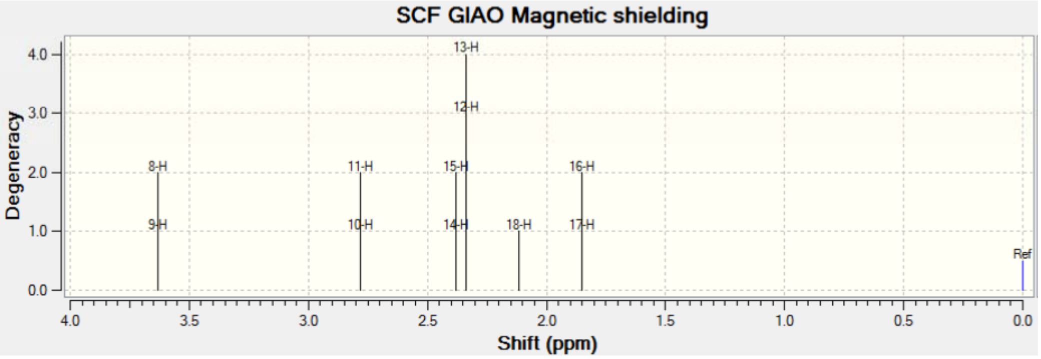

^{1}H NMR GIAO against reference TMS HF/6-31G(d) GIAO

Malono Nitrile

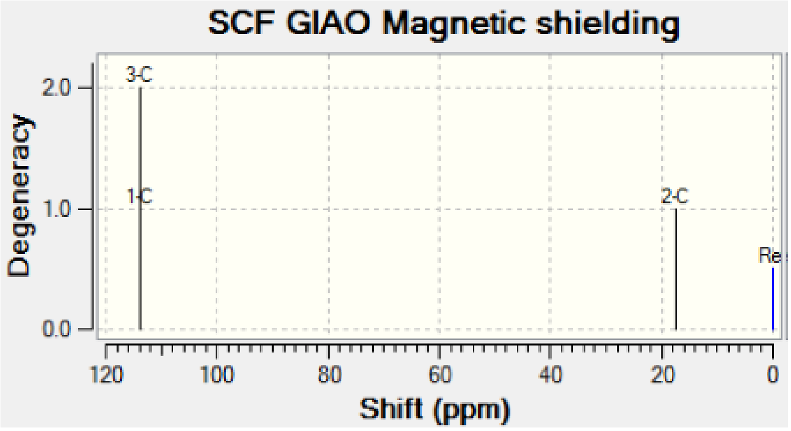

^{13}C NMR GIAO against reference TMS HF/6-31G(d) GIAO

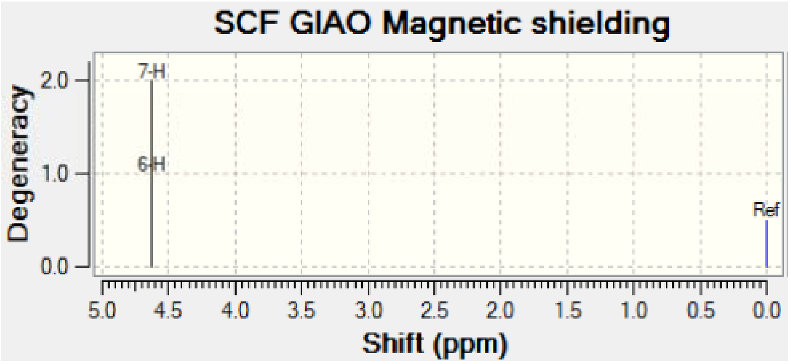

^{1}H NMR GIAO against reference TMS HF/6-31G(d) GIAO

Be—Malono Nitrile

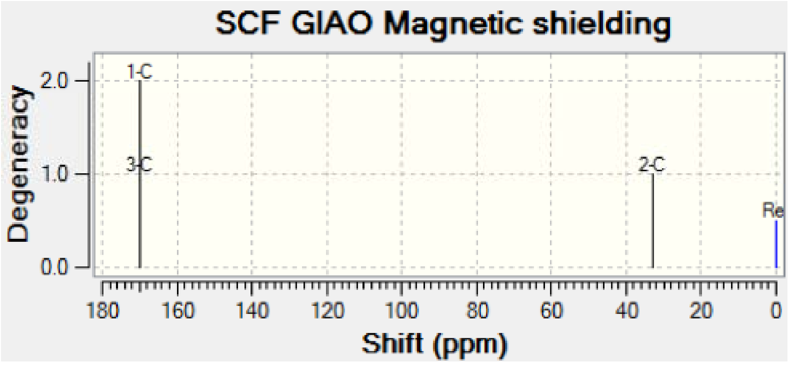

^{13}C NMR GIAO against reference TMS HF/6-31G(d) GIAO

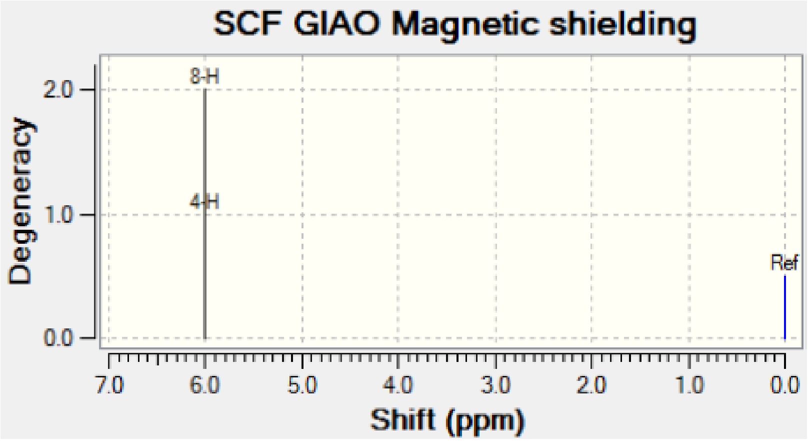

^{1}H NMR GIAO against reference TMS HF/6-31G(d) GIAO

5. Mulliken Charges

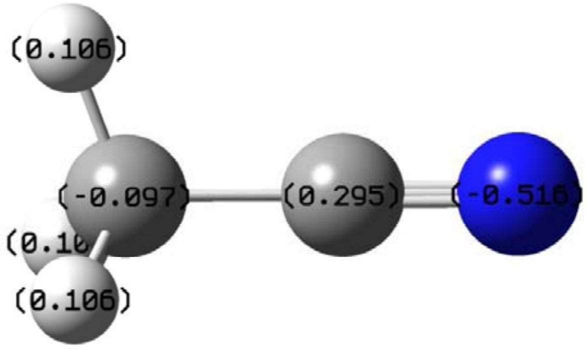

CH3CN

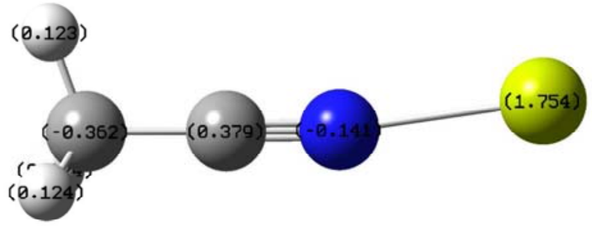

Mg-CH3CN

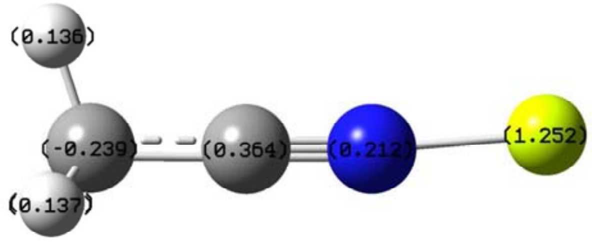

Be-CH3CN

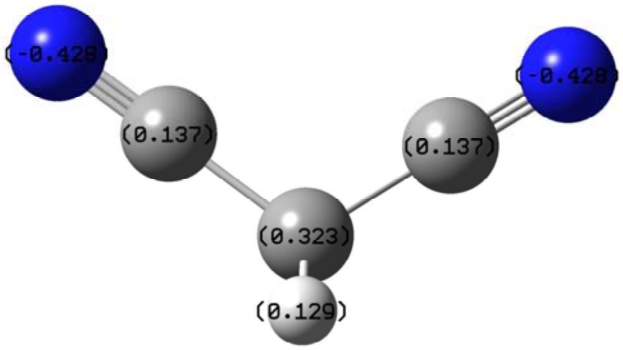

Malono nitrile

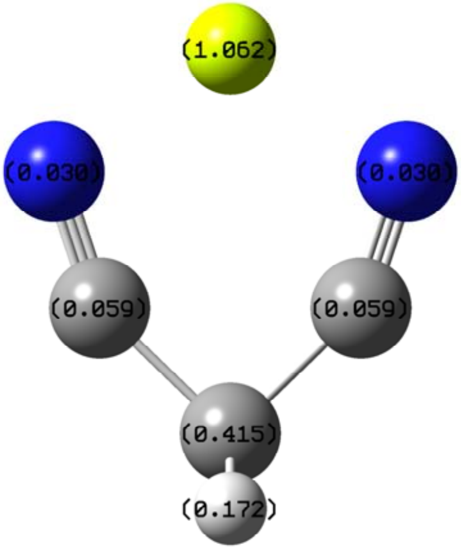

Be-Malono Nitrile

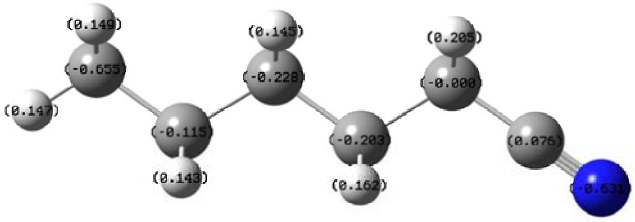

n-Pentane carbonitrile

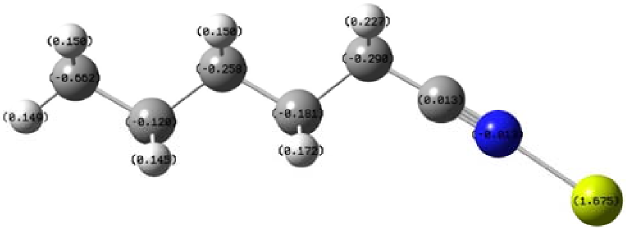

Mg--*n*-Pentane carbonitrile

7. Molecular Orbitals

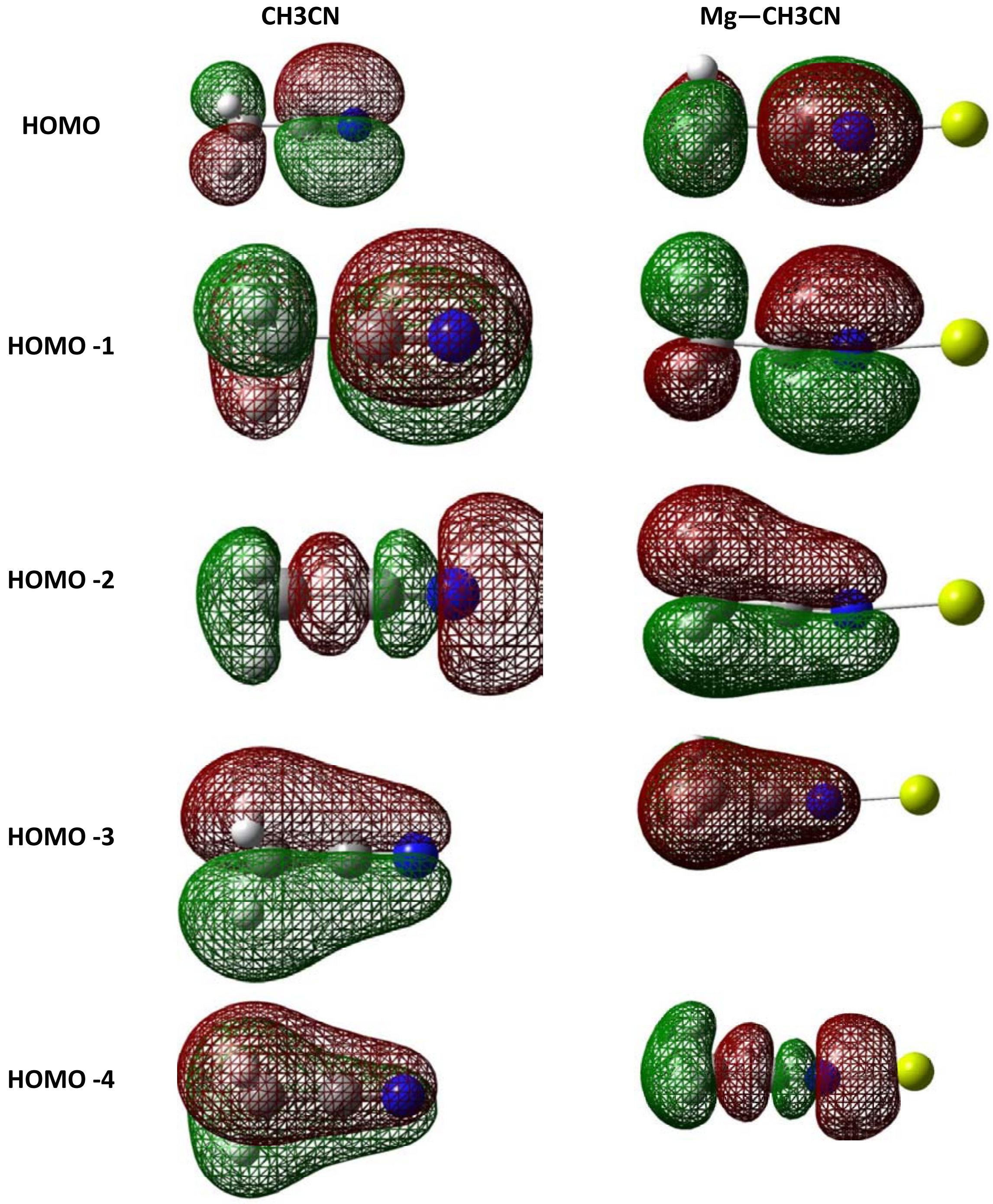

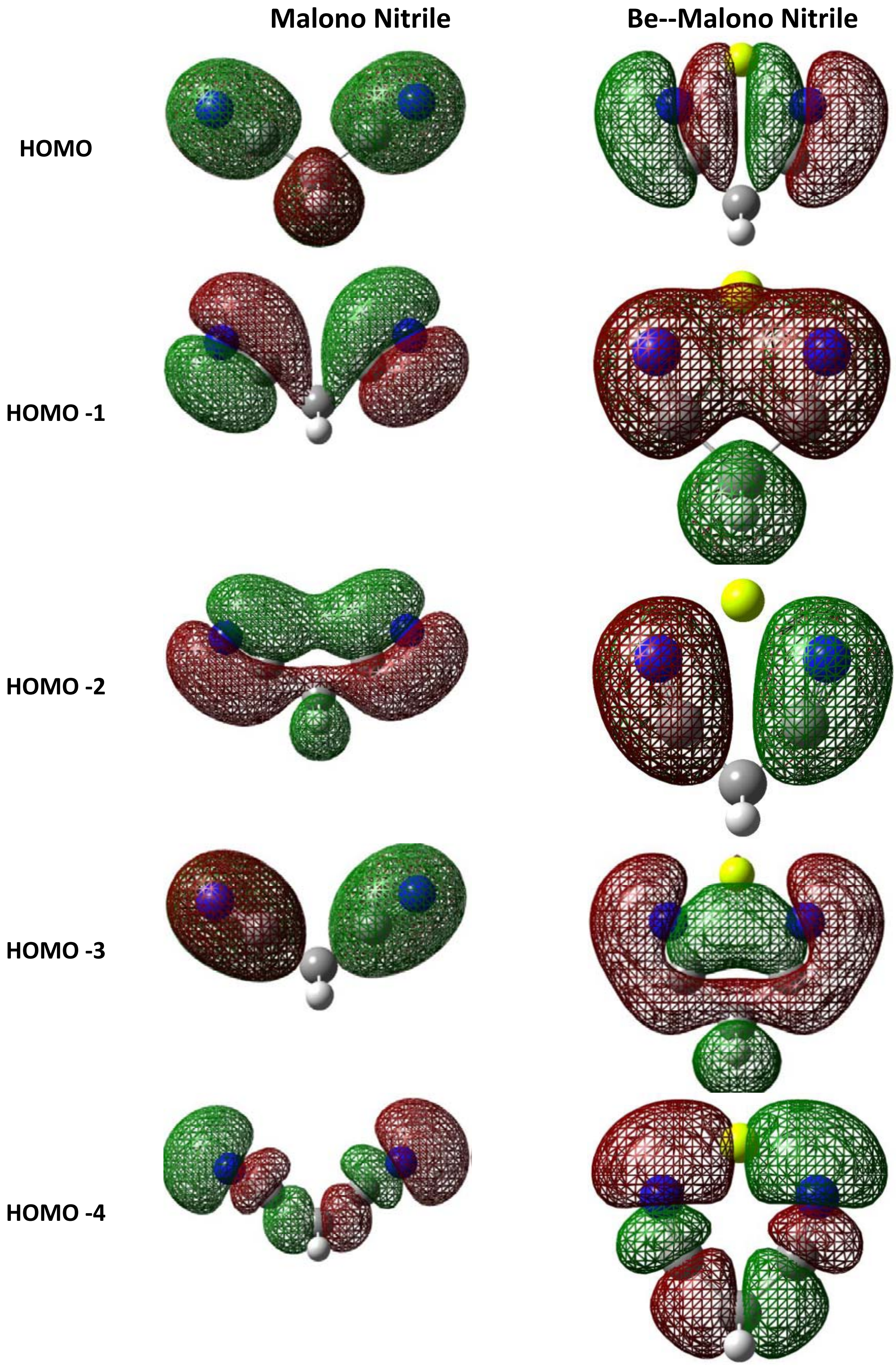
Malono Nitrile
Be--Malono Nitrile
HOMO
HOMO -1
HOMO -2
HOMO -3
HOMO -4

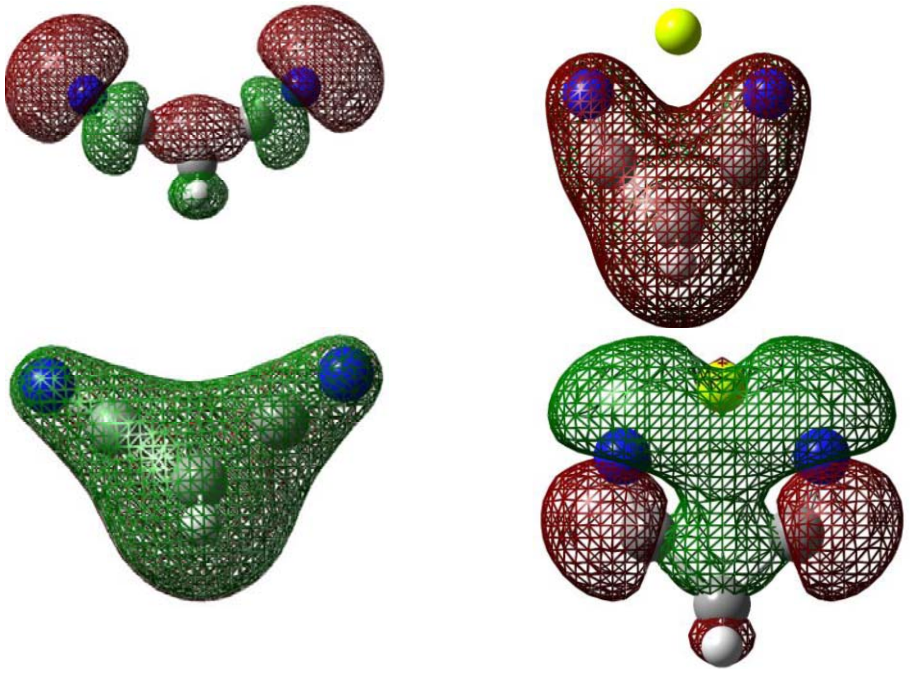

HOMO -5

HOMO -6

© 2013 by the authors; licensee MDPI, Basel, Switzerland. This article is an open access article distributed under the terms and conditions of the Creative Commons Attribution license (http://creativecommons.org/licenses/by/3.0/).
